# Pharmacognostic Characterization, Phytochemical Profiling, and In Vitro Biological Evaluation of *Zygophyllum fabago* L.

**DOI:** 10.3390/ijms27135907

**Published:** 2026-06-30

**Authors:** Ostemirkyzy Darika, Kapsalyamova Elmira, Daryono Hadi Tjahjono, Mombekov Serzhan, Ustenova Gulbaram, Kantureyeva Aigerim, Shulenova Gaukhar, Alimova Urziya, Zhaimbayeva Elmira, Botabayeva Rauan

**Affiliations:** 1 Department of Pharmaceutical Technology, School of Pharmacy, Asfendiyarov Kazakh National Medical University, Almaty 050012, Kazakhstan; 2 Department of Pharmacochemistry, School of Pharmacy, Bandung Institute of Technology, Bandung 40132, Indonesia; 3 Department of Engineering Disciplines of Good Practices, School of Pharmacy, Kazakh National Medical University, 88 Tole Bi Street, Almaty 050012, Kazakhstan; 4 Medical College, South Kazakhstan Academy of Medicine, Shymkent 160019, Kazakhstan

**Keywords:** antibacterial and antifungal activity, gas chromatography-mass spectrometry, macroscopy, microelements, microscopy, phytochemical analysis, *Zygophyllum fabago* L.

## Abstract

*Zygophyllum fabago* L. is traditionally used in folk medicine due to its antimicrobial, antifungal, and anti-inflammatory properties. The aim of this study was to conduct a comprehensive morphological, anatomical, phytochemical, and microbiological investigation of *Z. fabago* collected in the Zhetysu region of Kazakhstan and to evaluate the cytotoxic potential of its extract. Morphological and microscopic analyses of the aerial parts were performed to establish diagnostic characteristics of the plant material. A 70% ethanolic extract obtained by ultrasonic extraction was subjected to GC-MS and LC-MS analyses, quantitative phytochemical assessment, and elemental profiling. The extract was further fractionated using solvents of different polarity. Antimicrobial activity was evaluated against selected clinically relevant bacterial and fungal strains, while cytotoxicity was assessed using the *Artemia salina* model. Phytochemical analyses tentatively identified a diverse range of secondary metabolites, including flavonoids, sterols, triterpenoids, fatty acids, and other bioactive constituents, together with appreciable levels of phenolic compounds and mineral elements. Several solvent fractions exhibited antimicrobial activity against the tested microorganisms. In the *Artemia salina* assay, no detectable toxicity was observed for the ethanolic extract under the tested conditions. The results provide pharmacognostic and phytochemical data for *Z. fabago* collected in Kazakhstan and demonstrate the presence of diverse bioactive constituents. The observed antimicrobial activity and low toxicity in the applied screening model suggest that this species may represent a potential source of biologically active compounds and warrants further phytochemical, pharmacological, and toxicological investigations.

## 1. Introduction

*Zygophyllum fabago* L. (ZFL) is a perennial herbaceous species belonging to the family Zygophyllaceae, widely distributed in arid and semi-arid regions, including Central Asia, Iran, Syria, Turkey, Spain, China, and southern regions of Kazakhstan [1]. The species is traditionally recognized as a medicinal plant and has attracted scientific attention due to its diverse phytochemical composition.

Previous studies have reported that ZFL contains several classes of biologically active secondary metabolites, including flavonoids, triterpenoids, saponins, alkaloids, and phenolic compounds. These constituents are associated with a broad spectrum of biological activities, such as antibacterial, antifungal, anti-inflammatory, antioxidant, antiviral, and expectorant effects [2,3,4,5,6,7,8,9,10,11,12,13]. However, most available studies have focused on specific extracts or limited biological activities, and comprehensive phytochemical and pharmacognostic evaluations remain limited.

In particular, data on ZFL populations growing in Kazakhstan are scarce. Detailed information regarding the macroscopic and microscopic diagnostic characteristics of this species is also insufficient, which limits the development of standardized quality control and pharmacopoeial monographs. Furthermore, integrated studies combining pharmacognostic analysis with GC-MS and LC-MS-based phytochemical profiling, elemental composition, and biological evaluation of Kazakhstan populations are still lacking.

Environmental factors such as arid climate, soil salinity, and temperature fluctuations characteristic of Kazakhstan may strongly influence the biosynthesis and accumulation of secondary metabolites in plants. Therefore, investigation of geographically distinct populations of *Z. fabago* may provide important insights into its phytochemical variability and biological potential.

From a pharmacopoeial perspective, reliable diagnostic features, chemical characterization, and preliminary biological evaluation are essential for ensuring the quality and authenticity of medicinal plant raw materials. ZFL is listed among the medicinal plants of Kazakhstan flora and is considered a potential source of bioactive compounds [14,15,16]. However, herbal products with antifungal activity remain limited in the national pharmaceutical market, highlighting the need for further research into plant-derived antimicrobial agents [17,18,19,20].

To the best of our knowledge, a comprehensive pharmacognostic, phytochemical, and microbiological investigation of ZFL from the Zhetysu region of Kazakhstan has not been previously reported. Therefore, the present study aims to systematically investigate the morphological and anatomical characteristics, phytochemical composition (GC-MS and LC-MS), elemental profile, antimicrobial activity, and cytotoxic potential of ZFL.

The obtained results provide baseline data for the pharmacognostic characterization of ZFL and contribute to the existing knowledge of its chemical diversity and biological potential.

These findings may support future quality control, pharmacological, and phytochemical investigations of this species and contribute to the exploration of its bioactive natural compounds.

## 2. Results and Discussion

### 2.1. Macroscopic Characteristics

ZFL is a perennial herbaceous plant reaching approximately 30–75 cm in height with widely spreading, striped branches. The stems are erect, smooth, and the terminal shoots are directed upward. The plant flowers from April to June and demonstrates typical xeromorphic and succulent features that reflect its adaptation to arid growing environmental conditions.

**Figure 1:**
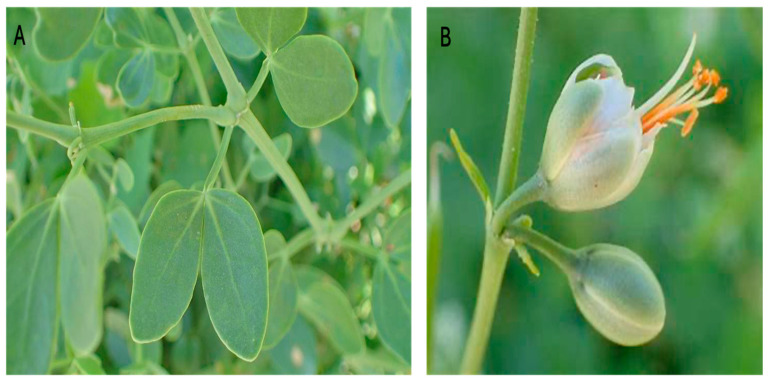
Macroscopic characteristics of ZFL: (**A**) leaves; (**B**) flower.

The leaves are opposite, bifoliate, and lack a rachis. The leaf surface is slightly convex, and the petiole is somewhat longer than the leaf blade. The paired leaflets are oval-elliptical with obovate shape and pinnate venation. The leaflet base is slightly oblique, while the apex is rounded (Figure 1A).

The flowers are borne on short peduncles and have a pale whitish coloration. The calyx consists of five ovate sepals with pale green coloration and a whitish margin. The petals are obovate with a blunt apex and a yellowish-red base. The stamens slightly exceed the petals in length, while the pistil consists of five angular carpels forming a multilocular ovary (Figure 1B).

The fruit is a drooping pentagonal capsule containing numerous seeds. The seeds are round-oblong and possess a characteristic shiny surface.

The observed succulent leaves, reduced leaf surface area, and thickened tissues are characteristic xeromorphic adaptations that enable the species to survive under drought and saline conditions commonly encountered in the arid regions of Kazakhstan. These morphological features may also influence the accumulation of secondary metabolites associated with stress adaptation.

### 2.2. Leaf Anatomy

Microscopic examination revealed anatomical features typical for xerophytic plants with C4 photosynthetic characteristics.

**Figure 2:**
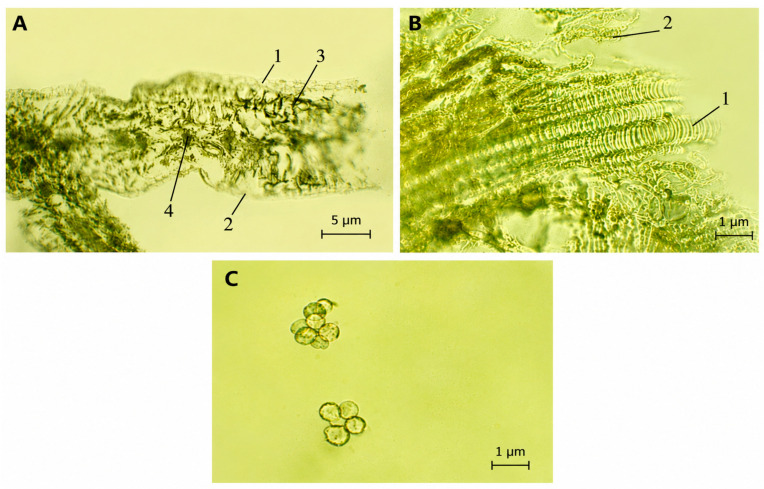
Microscopic characteristics of ZFL. (**A**) Anatomical structure of the ZFL leaf (×40): (1) upper epidermis, (2) lower epidermis, (3) mesophyll, and (4) vascular bundles. (**B**) Xylem vessels (×40): (1) xylem, (2) mesophyll; (**C**) drusen crystals in the mesophyll of the ZFL leaf (×40).

The leaf structure includes an upper and lower epidermis, mesophyll, and vascular bundles (Figure 2A). The epidermal cells are isodiametric with thickened outer walls. The leaf surface is amphistomatic, containing paracytic stomata on both sides of the leaf blade. The mesophyll consists of palisade-type chlorenchyma organized in several layers of elongated cells responsible for photosynthesis. Around the vascular bundles, cells rich in chloroplasts form a typical Kranz anatomical structure, which is characteristic of many plants performing C4 photosynthesis. Large water-storage cells are present in subepidermal layers on both sides of the leaf blade, contributing to drought resistance (Figure 2B). Numerous idioblasts containing drusen crystals are distributed within the mesophyll tissue (Figure 2C). The presence of these crystalline inclusions represents an important diagnostic pharmacognostic feature.

The presence of amphistomatic leaves, Kranz anatomy, and water-storage tissues confirms the adaptation of *Z. fabago* to xeric environments. Similar anatomical characteristics have been reported in other drought-tolerant members of the Zygophyllaceae family and may contribute to enhanced physiological resistance under environmental stress conditions.

### 2.3. Stem Anatomy

The stem of ZFL exhibits anatomical characteristics typical of xeromorphic plants and consists of the epidermis, cortical parenchyma, vascular cylinder, and central pith.

The epidermis is composed of a single layer of compact cells covered by a thick cuticle. Stomata are slightly immersed in the epidermal surface, which helps reduce water loss in arid environments.

The cortex consists mainly of parenchymatous tissue. In its outer region, several layers of chlorenchyma participate in photosynthetic activity. Beneath this layer, larger parenchymatous cells function as water-storage tissue, which represents an important adaptive feature.

**Figure 3:**
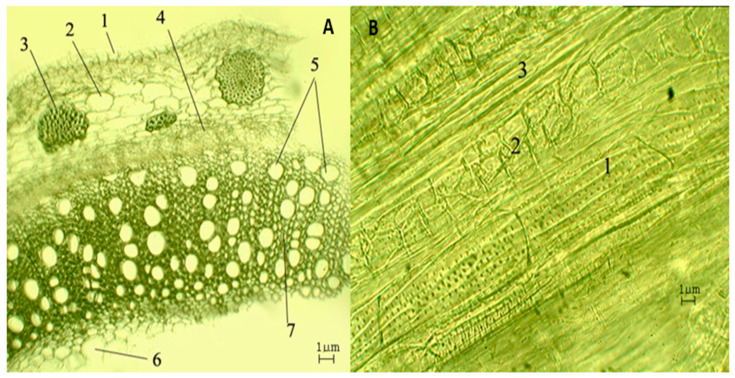
(**A**) Anatomical structure of the ZFL stem (×10): (1) epidermis, (2) cortex parenchyma, (3) sclerenchyma, (4) phloem, (5) xylem, (6) parenchyma cells, and (7) xylem fibres. (**B**) Longitudinal section of the stem of ZFL (×40): (1) xylem vessels, (2) xylem parenchyma cells, (3) xylem fibres.

Sclerenchymatous elements appear as discrete strands within the cortical tissue, providing mechanical support to the stem (Figure 3A). The vascular bundles are arranged in a ring-like pattern and have a collateral organization typical of species belonging to the Zygophyllaceae family.

The central region of the stem contains a well-developed parenchymatous pith. The xylem is composed of vessels, fibres, and radial parenchyma, while the phloem is located in the outer region of the vascular cylinder (Figure 3B).

The identified pharmacognostic characteristics of the leaf and stem anatomy provide reliable diagnostic features for the identification of ZFL as medicinal plant material.

The observed anatomical organization of the stem, including a well-developed vascular cylinder, parenchymatous cortex, and sclerenchymatous supporting tissues, reflects the xeromorphic adaptations of ZFL. The presence of water-storing parenchyma and protective epidermal structures contributes to the plant’s resistance to arid environmental conditions. These anatomical features represent important diagnostic characteristics for the pharmacognostic identification of this medicinal plant species.

From a pharmacognostic perspective, the combination of thick cuticle, water-storage parenchyma, collateral vascular bundles, and sclerenchymatous support tissues can be considered reliable diagnostic markers for the identification and quality control of ZFL raw material.

### 2.4. Phytochemical Analysis

A 70% ethanol extract of ZFL was obtained using ultrasonic extraction and subsequently subjected to gas chromatography-mass spectrometry (GC-MS) analysis, which resulted in the tentative identification of 51 compounds (Table 1). In addition to phytochemical profiling, the obtained extract was further utilized for quantitative analysis and for the determination of macro- and microelement contents.

GC–MS analysis revealed the presence of a wide range of secondary metabolites in ZFL including alkanes, alkenes, alcohols, terpenoids, carboxylic acids, phenolic derivatives, and esters. Among the tentatively identified compounds, hexadecanoic acid was one of the predominant constituents in ZFL samples collected in Kazakhstan, accounting for 8.22% of the total composition (Table 1). In comparison, previous studies have reported a higher content of this compound (14.38%) in ZFL samples collected in Western Azerbaijan [21].

Such regional variations in the concentration of hexadecanoic acid suggest that environmental factors, including soil characteristics, climatic conditions, and growing environments, may influence the formation and accumulation of secondary metabolites in medicinal plants. These findings highlight the importance of investigating geographically distinct populations in order to better understand the phytochemical variability of this species.

The predominance of fatty acids, long-chain hydrocarbons, and terpenoid derivatives identified by GC–MS may be relevant to the biological properties observed for several fractions of the extract. Similar classes of compounds have previously been reported to exhibit antibacterial and antifungal activities, including membrane-disrupting effects against microbial pathogens.

The detailed list of the tentatively identified compounds, including their chemical classes and reported biological activities, is presented in Table S1 (Supplementary Materials).

The GC–MS results also demonstrate substantial phytochemical diversity within the Kazakhstan population of ZFL, supporting the hypothesis that ecological and geographical factors may influence metabolite accumulation patterns.

Hexadecanoic acid has previously been reported to possess biological activity. Ganesan et al. demonstrated that n-hexadecanoic acid isolated from Ipomoea eriocarpa exhibited antibacterial activity against *Staphylococcus aureus*, *Bacillus subtilis*, *Escherichia coli*, and *Klebsiella pneumoniae*, producing inhibition zones ranging from 7.96 to 11.93 mm at a concentration of 50 μg/mL [22]. The detection of this compound in ZFL supports the potential biological relevance of the plant and justifies further investigation of its pharmacological properties.

However, the present study did not directly establish a causal relationship between individual compounds and the observed antimicrobial activity. Therefore, the contribution of specific metabolites should be considered preliminary and requires further confirmation through bioassay-guided isolation and mechanistic studies.

In addition to hexadecanoic acid, several other major compounds tentatively identified by GC–MS may also contribute to the overall biological profile of ZFL. Phytol has previously been reported to exhibit antibacterial, antifungal, antioxidant, and anti-inflammatory activities, whereas squalene is known for its antioxidant and membrane-protective properties. Octacosane and other long-chain hydrocarbons have likewise been associated with protective functions in plant defense systems.

Therefore, the antimicrobial activity observed in the dichloromethane and butanol fractions may be associated with the combined presence of multiple bioactive constituents. Nevertheless, the present study did not directly evaluate the contribution of individual compounds or possible synergistic interactions among them. Consequently, further bioassay-guided fractionation, compound isolation, and mechanistic investigations are required to determine the specific constituents responsible for the observed biological activities.

### 2.5. LC-MS Analysis Results

A 70% ethanol extract of ZFL was prepared using ultrasonic extraction and subsequently analyzed by liquid chromatography–mass spectrometry (LC-MS). As a result of the analysis, a total of 21 compounds were tentatively identified in the extract.

Compound annotation was performed based on accurate mass measurements, characteristic fragmentation patterns, retention behavior, and comparison with previously published LC-MS data. Since authentic reference standards were not analyzed, all compounds were considered tentatively identified (Metabolomics Standards Initiative, MSI level 2).

**Figure 4:**
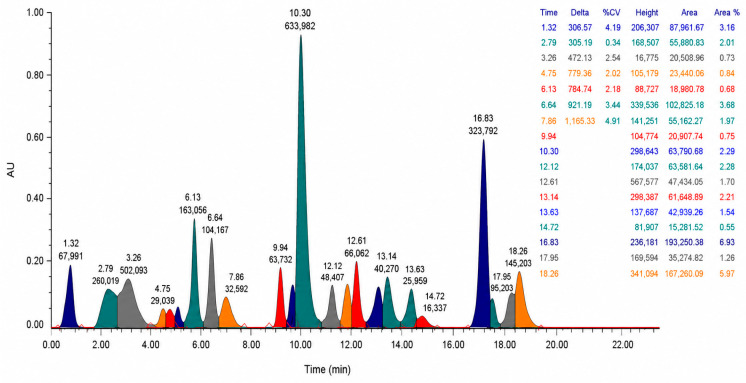
Chromatogram LC-MS analysis of ZFL Extract.

The tentatively identified compounds, their retention times, precursor ions, characteristic fragment ions, and relative peak areas are summarized in Table 2, while the corresponding chromatographic profile is presented in Figure 4.

The detailed mass spectra of the tentatively identified compounds are provided in the Supplementary Materials (Figures S1–S21), together with their corresponding retention times, precursor ions, and major fragment ions, to facilitate evaluation of the LC-MS identification process and avoid overloading the main text with extensive spectral data.

Liquid chromatography–mass spectrometry (LC-MS) analysis of the ZFL extract provided a detailed phytochemical profile, revealing a wide range of secondary metabolites, including flavonoids, terpenoids, organic acids, and other bioactive compounds. The data obtained allowed the tentative identification of several major constituents of interest based on their relative abundance and previously reported biological activities.

Chromatographic analysis revealed that acacetin was the most abundant tentatively identified compound in the extract, accounting for 28.63% of the total peak area with a retention time (RT) of 10.899 min. Acacetin is a flavone that has previously been reported to possess anti-inflammatory, antioxidant, and antitumor activities. Its relatively high abundance suggests that it may represent one of the major constituents of ZFL and could contribute to the biological properties associated with the extract.

A substantial proportion of the extract was also represented by β-sitosterol (14.65% at RT 16.833 min), a phytosterol that has been widely investigated for its potential health-promoting effects, including cholesterol-lowering activity. The relatively high abundance of this compound suggests that it may contribute to the overall biological profile of the extract.

Other notable compounds were detected at lower relative abundances. Oleanolic acid (7.12%), a triterpenoid, has been reported to exhibit hepatoprotective, anti-inflammatory, and antineoplastic activities. Similarly, the flavonoids quercetin (4.69%) and kaempferol (4.29%) are widely recognized for their antioxidant and anti-inflammatory properties. The occurrence of these metabolites further demonstrates the chemical diversity of the ZFL extract.

Additionally, β-amyrin (4.32%), another triterpenoid, together with phenolic acid derivatives such as chlorogenic acid and sinapinic acid, was detected in the extract. The combined presence of these metabolites may contribute to the overall biological profile of the extract. However, possible synergistic interactions among these constituents were not evaluated in the present study and therefore remain speculative.

Taken together, the LC-MS results provide a valuable basis for further phytochemical and pharmacological investigations of ZFL. The relatively high abundance of acacetin and β-sitosterol, together with the presence of other biologically relevant metabolites such as oleanolic acid, quercetin, kaempferol, and β-amyrin, indicates that the extract is a chemically diverse source of potentially bioactive natural products. Future studies should focus on the isolation of individual compounds and evaluation of their specific biological activities.

Several of the tentatively identified flavonoids, including acacetin, quercetin, kaempferol, isorhamnetin, and apigenin, have previously been reported to possess antimicrobial and antioxidant activities. However, the present study did not directly establish a relationship between individual compounds and the observed biological activities of the extract. Therefore, the contribution of specific metabolites should be considered preliminary and requires confirmation through bioassay-guided isolation and mechanistic studies.

The comparative analysis demonstrates the importance of combining LC-MS and GC-MS for comprehensive phytochemical characterization of complex plant extracts. LC-MS enabled the characterization of predominantly polar metabolites, including flavonoids, phenolic acids, triterpenoids, and phytosterols, whereas GC-MS provided complementary information on less polar constituents such as hydrocarbons, fatty acids, and long-chain alcohols. The integration of both analytical approaches provided a more comprehensive understanding of the phytochemical diversity of ZFL and highlighted the complexity of its chemical composition.

### 2.6. Quantitative Analysis of Bioactive Compounds

Table 3 provides the quantitative analysis results for key bioactive compounds, including extractive substances, flavonoids, alkaloids, tannins, saponins, polysaccharides, coumarins, and organic acids.

The ZFL extract from Kazakhstan contained 15.419% extractive substances, 5.574% tannins, and 2.775% coumarins. This profile is consistent with the data of Yaripour et al. [2] and Abdel-Hamid et al. [16], who reported comparable tannin and coumarin contents in ZFL extracts. Notably, the tannin concentration in the Kazakhstan sample was higher than in samples obtained from other regions, suggesting that the specific environmental conditions of Kazakhstan may influence the activation of tannin biosynthesis. Such differences highlight the importance of geographic factors in shaping the phytochemical composition of plant materials and may explain the variability in their pharmacological properties.

Abdel-Hamid et al. [14] also recorded the presence of flavonoid glycosides, phenolic acids, and terpenoids in 70% ethanol extracts of ZFL, while free flavonoids and carbohydrates were additionally detected in 50% ethanol and ethyl acetate extracts. In their study, organic acids, including p-coumaric, vanillic, and ferulic acids, accounted for 7.96% of the total organic acids. In contrast, the concentration of organic acids in the ZFL extract from Kazakhstan was only 0.225%. This discrepancy likely reflects the influence of climatic conditions, soil composition, and other environmental factors on plant metabolic pathways. This confirms the presence of notable regional differences in the phytochemical profiles of ZFL, determining their potential as a source of bioactive compounds.

The relatively high contents of tannins and coumarins detected in the Kazakhstan samples may be particularly important for the antimicrobial properties of the extract, as both classes of compounds have previously been reported to inhibit the growth of various bacterial and fungal microorganisms.

### 2.7. Macroelement and Microelement Composition

Elemental analysis was performed to support pharmacognostic quality assessment and to provide preliminary information regarding the safety and mineral composition of the investigated plant material.

Macroelement and microelement analyses revealed high concentrations of calcium, potassium, sodium, and magnesium in the ZFL sample (Table 4). The Kazakhstani ZFL also displayed trace levels of elements such as iron and zinc, which are essential for various cellular processes.

Quantitative analysis of ZFL samples revealed that the plant contained 10.5% moisture and 18.88% total ash. The elevated ash content may be due to the accumulation of considerable amounts of macro- and micronutrients, with the highest concentrations recorded for calcium (520.31 mg/kg DW), potassium (601.91 mg/kg DW), and sodium (362.62 mg/kg DW). In comparison, Abdel-Hamid et al. reported lower moisture (8.26%) and ash (5.43%) values, as well as markedly lower concentrations of sodium (174.17 mg/kg DW), potassium (72.73 mg/kg DW), and calcium (33.70 mg/kg DW) in ZFL extracts.

These differences may indicate the plant’s ability to accumulate inorganic elements depending on environmental conditions. This accumulation may play a role in strengthening antioxidant defenses and also reflect the organ-specific response of ZFL to the presence of heavy metals and other environmental stressors [26]. Thus, the identified regional differences highlight the importance of eco-geographical factors in the formation of the mineral and physiological profile of the plant.

The absence of detectable lead and the very low concentration of cadmium indicate that the collected plant material meets basic safety expectations regarding heavy-metal contamination. This observation is important for the future development of pharmacognostic standards for this species.

### 2.8. Preparation of ZFL Fractions and Antimicrobial Activity

Various ZFL fractions (petroleum ether, dichloromethane, ethyl acetate, butanol, and water) were prepared for microbiological assays (Table 5).

Antibacterial analysis revealed that all tested ZFL fractions exhibited activity against *S. aureus*, with the dichloromethane fraction producing the largest inhibition zone. Against *E. coli*, the butanol fraction showed the highest inhibitory effect. In contrast, the aqueous fraction showed no activity against *E. faecalis*, while the petroleum ether and dichloromethane fractions exhibited no detectable activity against *P. aeruginosa*.

The inhibition-zone diameters obtained for each fraction against the tested microorganisms are presented in Table 6. The observed differences among fractions indicate that antimicrobial activity is influenced by solvent polarity and the distribution of bioactive constituents within the extract.

Although several fractions demonstrated measurable inhibitory effects, the inhibition zones were generally modest and, in some cases, close to those observed for the controls. Therefore, these findings should be considered preliminary screening data indicating potential antimicrobial activity rather than definitive evidence of strong antimicrobial efficacy.

The comparatively higher activity observed for the butanol and dichloromethane fractions suggests that moderately polar constituents may contribute to the antimicrobial properties of ZFL. However, further studies involving isolation of active compounds and additional microbiological assays are required to confirm these observations.

For comparison, Abdel-Hamid et al. [16] demonstrated that the n-hexane fraction of ZFL extract possessed antifungal activity against *C. albicans*, producing an inhibition zone of 9.5 mm. In our study, the butanol fraction exhibited a more pronounced inhibitory effect (14.3 ± 1.1 mm) against *C. albicans*, followed by the petroleum ether fraction (11 ± 1.2 mm) and the aqueous fraction (10.6 ± 1.1 mm) (Table 7). These differences in activity highlight the importance of the choice of extraction method and solvent, as they substantially affect the concentration and composition of the biologically active components that determine the antimicrobial potential of ZFL fractions.

Additionally, Zaidi et al. [27,28,29] demonstrated that ZFL extracts were capable of inhibiting up to 95% of *C. albicans* growth at a concentration of 200 μg/mL, which is consistent with the results of our study, where the butanol and petroleum ether fractions showed notable antifungal activity in the preliminary screening assay.

Further support for the antifungal potential of ZFL is provided by the data of Dana et al. [30] who demonstrated that an aqueous extract of ZFL effectively inhibited the growth of *Fusarium oxysporum* f. sp. *melonis* and *Pythium aphanidermatum* with inhibition rates of 42.9% and 85.3%, respectively.

Taken together, these data indicate a broad spectrum of antifungal activity for ZFL extracts, capable of inhibiting a variety of pathogenic fungi. The observed variations in efficacy between studies highlight the critical role of solvent choice and extraction method in maximizing the therapeutic potential of ZFL, as each solvent extracts specific bioactive compounds responsible for activity against specific fungal pathogens.

The results obtained in the present study support the traditional use of ZFL in folk medicine for the treatment of infectious conditions and justify further bioactivity-guided fractionation studies aimed at identifying the compounds responsible for the observed antimicrobial effects.

### 2.9. Minimum Inhibitory Concentration (MIC) Test Results

The MIC values for each fraction against tested microbes are summarized in Table 8. Most fractions had MIC values above 1000 µg/mL, with notable exceptions: the ethyl acetate fraction exhibited notable inhibition against *E. faecalis* (500 µg/mL), *S. aureus* (1000 µg/mL), and *P. aeruginosa* (250 µg/mL). The butanol fraction showed efficacy against *C. albicans* (1000 µg/mL), *M. furfur* (250 µg/mL), and *A. niger* (250 µg/mL), while the water fraction effectively inhibited *P. aeruginosa* (500 µg/mL).

Previously, Alamholo et al. [31] reported that methanolic extracts of ZFL exhibited activity against *E. faecalis*, with root (50 μg/mL) and flower (25 μg/mL) extracts identified as the key active components. In contrast, in our study, activity against *E. faecalis* was detected only in the ethyl acetate fraction at a concentration of 500 μg/mL, while other fractions required concentrations exceeding 1000 μg/mL to achieve comparable inhibitory effects.

This difference highlights the critical role of solvent selection and extraction method in determining the antimicrobial activity of ZFL. The results suggest that specific fractions may contain higher concentrations of the bioactive compounds responsible for inhibiting microbial growth. These observations confirm that optimization of the extraction method is key to maximizing the therapeutic potential of ZFL.

According to commonly accepted criteria for plant extracts, fractions exhibiting MIC values below 1000 μg/mL may be considered biologically active. Therefore, the ethyl acetate, butanol, and aqueous fractions demonstrated noteworthy antimicrobial potential and warrant further phytochemical investigation.

### 2.10. Cytotoxicity Assay

The results of the cytotoxic activity studies are presented in Table 9. The ethanolic extract of ZFL did not exhibit cytotoxic effects in the *Artemia salina* lethality assay.

No mortality was observed in *Artemia salina* larvae exposed to the ethanolic extract at concentrations up to 10 mg/mL, indicating the absence of acute toxicity under the experimental conditions. According to the *Artemia salina* bioassay, extracts that do not induce substantial larval mortality are generally considered to possess low toxicity in this preliminary screening model.

In contrast, the reference compound Actinomycin D demonstrated pronounced cytotoxicity toward *Artemia salina* larvae at all tested concentrations, resulting in mortality rates ranging from 74% to 96%.

Although the extract did not exhibit detectable toxicity in the *Artemia salina* assay, these results should be considered preliminary. Additional studies using mammalian cell lines and in vivo toxicity models are necessary to further evaluate the safety profile of *Z. fabago* extracts.

## 3. Materials and Methods

### 3.1. Macroscopy and Microscopy

Following Good Manufacturing Practice (GMP) guidelines, the production process begins with thorough cleaning of equipment, area, and attire to prevent microbial contamination [32,33]. The ZFL plant was collected in the Zhetysu region, Panfilovsky district, near the village of Koktal (GPS coordinates 44.145022, 79.793069), in accordance with Good Agricultural and Collection Practice (GACP) in May and June 2023. The plant material was dried in a room at a temperature of 25 ± 5 °C with ventilation. Once dried, the material was crushed to a size of 1–3 mm using an IKA M20 laboratory mill (IKA-Werke GmbH & Co. KG, Staufen, Germany). The plant reserves in this region were identified on the basis of research by the Laboratory of Plant Biomorphology of Al-Farabi Kazakh National University.

For the anatomical study of ZFL, a dried stem of the plant was fixed in 70% alcohol solution (alcohol/glycerin/water, 1:1:1). A cross-section of the stem, 10–15 μm thick, was made with a TOS-2 freezing microtome ((MICROS, Produktions- und Handels GmbH, Vienna, Austria). Anatomical images to identify the morphological features of the stem were taken with an MS-300 binocular microscope (MICROS, Produktions- und Handels GmbH, Vienna, Austria). General terminology was used when describing the anatomical features [34,35,36,37,38,39].

### 3.2. Ultrasonic Extraction of the ZFL Herb

Following Gharibzahedi and Altintas’s modified ultrasonic extraction method [40], ZFL aerial parts (50 g) were subjected to 70% ethanol (500 mL) extraction using a KQ5200B ultrasonic apparatus (KSMM, Kunshan Ultrasonic Instruments Co., Ltd., Kunshan, China) at 40 kHz for 1 h. The extract was filtered and concentrated at 45 °C.

A 70% ethanol solution was selected as the extraction solvent because hydroalcoholic mixtures are widely used in phytochemical investigations due to their ability to extract both polar and moderately non-polar plant metabolites. Such solvent systems are particularly effective for the recovery of flavonoids, phenolic compounds, terpenoids, fatty acids, and other secondary metabolites. Therefore, 70% ethanol was chosen as a universal solvent to obtain a representative phytochemical profile of *Zygophyllum fabago* L. in the present study.

### 3.3. Determination of the Moisture Content of ZFL

The moisture content was measured using a modified method of Zambrano et al. [41]. The dried ZFL herb (1 g) was weighed in a predried weighting bottle and dried in an oven at 100–105°C for 30 min. The bottle was weighed, and the procedure was repeated until a constant weight was achieved (the difference between the last two weights did not exceed 0.1 g). The moisture content (*X*) of the raw material was calculated with the following Equation (1) [41]:

(1) $$X=\frac{M-M_{1}}{M}\times 100$$

where *M* is the initial weight of the raw material (g), and *M*_1_ is the weight after drying (g).

### 3.4. Determination of the Mineral Composition of ZFL

The mineral composition of ZFL was determined according to a modified method of Kantureyeva et al. [42,43]. Approximately 1 g of ZFL preparation (or 3–5 g of dried herb) was placed in a pre-calcined porcelain crucible and subjected to calcination until complete combustion of organic matter was achieved.

The resulting residue was dissolved in 5 mL of HNO_3_ solution (1:1) with heating and further diluted with either 1 N HCl or 1 N HNO_3_. The solution was transferred to a 25 mL volumetric flask and diluted to volume.

A blank solution was prepared under the same conditions. Mineral elements were determined using an ASSIN atomic absorption spectrometer (Carl Zeiss, Jena, Germany).

### 3.5. Determination of Total ZFL Ash Content

The total ash content of ZFL was determined according to a modified method of Momin and Kadam [44]. Approximately 1 g of ZFL preparation (or 3 g of dried herb) was placed in a pre-weighed crucible and subjected to calcination in a muffle furnace at about 500 °C until constant weight was obtained.

After cooling in a desiccator, the crucible was weighed. The percentage of total ash (*X*) in the absolutely dry raw material was calculated using Equation (2):

(2) $$X=\frac{m\times 100\times 100}{M_{1}\times (100-W)}$$

where *m* is the weight of the residue (g), *M*_1_ is the weight of the raw material (g), and *W* is the moisture content of the raw material (%).

### 3.6. Quantitative Determination of Organic Acids

The quantitative determination of free organic acids was conducted using a modified method of Fras et al. [45]. Carboxylic acids were quantified by titration. The dried ZFL herb (10 g) was weighed into a 100 mL flask, and 80 mL of distilled water was added. The mixture was then heated in a boiling water bath for 2 h, cooled, and filtered into a 100 mL volumetric flask, with distilled water added to reach the final volume, creating a standard solution.

To a portion of the standard solution (10 mL) in a 100 mL flask, 1 mL of 1% alcoholic phenolphthalein solution was added, and the solution was titrated with 0.1 M sodium hydroxide solution until a pale pink color appeared.

The content of free organic acids (*X*%) in the completely dry raw material was calculated using Equation (3):

(3) $$X=\frac{V\times P\times 25\times 100\times 100}{m\times 10\times (100-W)}$$

where *V* is the volume of 0.1 M sodium hydroxide solution used for titration (mL), *m* is the weight of raw material (g), *W* is the loss on drying of the raw material (%), and *P* refers to malic acid (0.0067 g) or valeric acid (0.01021 g).

### 3.7. Quantitative Determination of Flavonoids in Terms of Quercetin

The quantitative determination of flavonoids in terms of quercetin was carried out based on the modified method of Aparna and Hema [46]. 1 g of dried ZFL herb was weighed and placed in a 150 mL flask fitted with a reflux condenser. Then, 30 mL of 90% ethanol containing either 1% concentrated HCl or 10% H_2_SO_4_ (for hydrolysis of glycosides) was added, and the mixture was heated in a boiling water bath for 1 h. After cooling to room temperature, the mixture was filtered through a paper filter into a 100 mL volumetric flask. This procedure was repeated twice, after which the filter was washed with 90% ethanol, and the combined filtrates were diluted to volume with 90% ethanol to provide solution A.

To 2 mL of solution A in a 25 mL volumetric flask, 1 mL of 1% AlCl_3_ in 95% ethanol was added, and the solution was diluted to volume with 95% ethanol. After 20 min, the absorbance of the solution was measured at a wavelength of 430 nm in a cuvette with a path length of 10 mm using a spectrophotometer. For comparison, 2 mL of solution A diluted with 90% ethanol in a 25 mL volumetric flask was used. The flavonoid content (*X*%) in the absolutely dry raw material, in terms of quercetin, was calculated using Equation (4):

(4) $$X=\frac{D\times 100\times 100\times 25}{764.6\times m\times 2\times (100-W)}$$

where D is the absorbance of the test solution at 430 nm, 764.6 is the specific absorption index of the quercetin complex with 1% aluminum chloride at 430 nm, *W* is the percentage loss during drying of the raw material, and *m* is the weight of the raw material (g).

### 3.8. Quantitative Determination of Polysaccharides

The quantitative determination of polysaccharides was conducted using a modified method of Hu et al. [47]. The dried ZFL herb (5 g) was weighed into a 100 mL flask fitted with a reflux condenser. Distilled water (50 mL) was added, and the mixture was heated in a boiling water bath for 1 h. After cooling and filtration, the extraction procedure was repeated twice under the same conditions for 30 min each time. The aqueous extracts were combined and filtered into a 250 mL volumetric flask through three layers of gauze. The filter was washed with distilled water and diluted to volume with distilled water to create solution B.

To solution B (25 mL) in a centrifuge tube, 95% ethanol (75 mL) was added, and the mixture was heated in a water bath at 60 °C for 5 min. After 30 min, the contents were centrifuged at a speed of 5000 rpm for 30 min. The supernatant was filtered under vacuum through a VitraPOR 16 glass filter and dried to a constant weight. The precipitate was then quantitatively transferred to the same filter and washed with 95% ethanol (15 mL). The filter with the precipitate was dried at 100–105 °C to a constant weight.

The Content of Polysaccharides (*X*%) in the Dry Raw Material Was Calculated Using Equation (5):

(5) $$X=\frac{\left(m_{2}-m_{1}\right)\times 250\times 100\times 100}{m\times 25\times (100-W)}$$

where *m*_1_ is the weight of the filter (g), *m*_2_ is the weight of the filter with residue (g), *m* is the weight of the raw material sample (g), and *W* is the loss on drying of the raw material (%).

### 3.9. Permanganatometric Determination of Tannins

The permanganatometric determination of tannins was conducted using a modified method of Hamzah et al. [48]. The crushed raw material (1 g) was weighed into a 100 mL conical flask, hot distilled water (50 mL) was added, and the mixture was heated in a boiling water bath for 2 h. The aqueous phase was decanted with minimal loss, and the extraction procedure was repeated. The combined extracts were filtered into a 100 mL volumetric flask and diluted to volume with distilled water to create solution C.

Distilled water (100 mL) and indigosulfonic acid solution (10 mL) were added to solution C (10 mL) in a 500 mL conical flask. The solution was then titrated with constant stirring using a 0.02 M potassium permanganate solution until a golden yellow color appeared. In a parallel control experiment, an indigosulfonic acid solution (10 mL) in distilled water (100 mL) was titrated.

The potassium permanganate solution (0.02 M, 1 mL) corresponds to 0.004157 g of hydrolysable tannins or 0.00582 g of condensed tannins in terms of a reference tannin. The content of tannins (*X*%) in the dry raw material was calculated using Equation (6):

(6) $$X=\frac{(V_{1}-V_{2})\times K\times D\times V\times 100}{V_{3}\times m\times (100-W)}$$

where *V*_1_ is the volume of 0.02 M potassium permanganate solution used for extract titration (mL), *V*_2_ is the volume of 0.02 M potassium permanganate solution used in the control experiment (mL), *V*_3_ is the volume of extract taken for titration (mL), *V* is the total volume of the extract (mL), *m* is the weight of raw material (g), *W* is the loss on drying of the raw material (%), and D is the conversion factor to the corresponding tannins.

### 3.10. Quantitative Determination of Coumarins

The quantitative determination of coumarins was conducted using a modified method of Lončar et al. [49]. The milled, dried ZFL herb (2 g) was weighed into a 100 mL flask and extracted with chloroform (3 × 25 mL). The combined chloroform extracts were evaporated to dryness, redissolved in 96% ethanol (10 mL), transferred to a 25 mL volumetric flask, and diluted to volume with 96% ethanol to create solution D. Solution D (1 mL) was then diluted to volume in a 50 mL volumetric flask with 96% ethanol to provide solution E. The absorbance of solution E was measured in a 10 mm path length cuvette at 272 nm, with 96% ethanol used as the reference solution. The content of the sum of coumarin derivatives (*X*%) was calculated using Equation (7) [49]:

(7) $$X=\frac{D\times 25\times 50\times 100\times 100}{734\times m\times 10\times 1(100-W)}$$

where D is the absorbance of solution E at 272 nm, *m* is the weight of the raw material (g), and *W* is the moisture content of the raw material (%).

### 3.11. Quantitative Determination of Saponins Content

The quantitative determination of saponins was conducted using a modified method of Chua et al. [50]. The milled, dried ZFL herb (2 g) was weighed into a 100 mL flask, and 20 mL of 3% nitric acid solution in acetone was added. The mixture was stirred for 1 h and then filtered. The solid residue was extracted three additional times with 20 mL of acetone, each time heating to reflux in a water bath for 30 min. All acetone extracts were combined in a 200 mL beaker, along with ethanol (10 mL), which was used to rinse all glassware. A concentrated ammonia solution was added dropwise with vigorous stirring until a plentiful, light yellow curd precipitate formed (pH 8.3−8.6, where wet phenolphthalein paper developed pink). The precipitate was separated on a Buchner funnel, and the beaker and filter cake were washed 2–3 times with acetone (a total of 30 mL). The dry filter cake was redissolved in distilled water (50 mL), quantitatively transferred into a 100 mL volumetric flask, and diluted to volume with distilled water to provide solution F. The absorbance of solution F in a cuvette with a path length of 10 mm was measured at 258 nm, using purified water as the reference solution.

The content of saponins (*X*%) in the dry raw material, expressed as glycyrrhizic acid, was calculated using Equation (8) [51]:

(8) $$X=\frac{D\times 822\times 100\times 100}{11000\times m\times (100-W)}\times 100$$

where D is the absorbance of the test solution at 258 nm, 11,000 is the specific ab-sorption index of the glycyrrhizic acid solution at 258 nm, 822 is the molecular weight of glycyrrhizic acid, *m* is the weight of the raw material (g), and *W* is the loss on drying of the raw material (%).

### 3.12. Quantitative Determination of Alkaloids Contents

The quantitative determination of alkaloids was performed using a modified method of Selvakumar et al. [52]. The dried ZFL herb (10 g) was weighed into a 250 mL flask, and then chloroform or ethyl acetate (100 mL) and concentrated ammonia solution (5 mL) were added. The mixture was shaken on a vibratory shaker for 2 h (or alternatively, left at room temperature for 15 h, followed by shaking for 30 min). The mixture was then filtered through cotton wool. A portion of the filtrate (50 mL) was concentrated in a 100 mL flask to 1–2 mL, with residual chloroform removed by blowing air over the solution. Sodium hydroxide solution (0.1 M, 2 mL) was added, and lumps were triturated with a glass rod. Distilled water (8 mL) was added, and the mixture was stirred for 2–3 min. A 0.1 M hydrochloric acid solution (10 mL) was added, followed by incubation for 8–10 min with shaking on a vibratory shaker. The mixture was then filtered through a triple paper filter with a diameter of 7 cm. To a 10 mL portion of the filtrate in a 50 mL flask, distilled water (10 mL) and two drops of methyl red solution were added. The excess acid was titrated with 0.01 M sodium hydroxide solution until a yellow color appeared.

In a parallel control experiment, 1 mL of 0.1 M sodium hydroxide solution in a 50 mL flask was combined with 4 mL of distilled water and 5 mL of 0.2 M hydrochloric acid. Two drops of methyl red solution were added, and the excess acid was titrated with 0.1 M sodium hydroxide solution until a yellow color appeared.

The content of the sum of the alkaloids (*X*%) in terms of the thermopsine in the completely dry raw material was calculated using Equation (9) [52]:

(9) $$X=\frac{\left(V_{1}-V_{2}\right)\times 0.0244\times 4\times 100\times 100}{m\times (100-W)}$$

where 0.0244 represents the amount of alkaloids (g) in terms of thermopsine, corresponding to a hydrochloric acid solution (0.1 M, 1 mL); *V*_1_ is the volume of sodium hydroxide solution (0.1 M) used for the control titration (mL); *V*_2_ is the volume of sodium hydroxide solution (0.1 M) used for titration of the test solution (mL); *m* is the weight of the raw material (g); and *W* is the loss on drying of the raw material (%).

### 3.13. Determination of Organic Compounds in the ZFL Extract via GC-MS

The determination of organic compounds in the ZFL extract by GC-MS was performed according to a modified method of Osman et al. [53].

Separation was carried out using a DB-WaxExt chromatographic capillary column (30 m × 0.25 mm × 0.25 μm, (Agilent Technologies, Santa Clara, CA, USA) with helium as the carrier gas at a constant flow rate of 1 mL/min. The oven temperature was programmed from 40 °C to 280 °C at a rate of 5 °C/min and maintained at the final temperature for 5 min (total run time: 53 min). The injection volume was 0.5 μL, the injector temperature was 250 °C, and analyses were performed in splitless mode. Mass spectra were acquired in scan mode over the *m*/*z* range 34–750. Data acquisition and processing were performed using Agilent MSD ChemStation software (version 1701EA).

Compound annotation was based on comparison of the obtained mass spectra with those available in the Wiley 7th Edition and NIST 02 mass spectral libraries. The “Similarity (%)” values reported in Table 1 represent the degree of agreement between the experimental mass spectrum and the corresponding reference library spectrum. Higher similarity values indicate better agreement and higher confidence in tentative compound annotation. Since authentic reference standards were not used, all GC-MS compounds are reported as tentatively identified.

### 3.14. Determination of Organic Compounds in the ZFL Extract via LC-MS

The determination of organic compounds in the ZFL extract by LC-MS was performed according to a modified method of Tu et al. [54].

The ZFL extract was dissolved in LC-MS grade methanol and analyzed using an Ultra Performance Liquid Chromatography (UPLC) system coupled to a Xevo G2-S QTOF-MS mass spectrometer (Waters Corporation, Milford, MA, USA) equipped with an electrospray ionization (ESI) source. Distilled water (A) and methanol (B) were used as the mobile phases. A 0.3 μL aliquot of the extract was injected onto an ACQUITY UPLC HSS C18 column (1.8 μm, 2.1 × 100 mm). LC-MS/MS chromatograms and mass spectra were processed and interpreted using MassLynx V4.1 software.

Compound annotation was performed using accurate mass measurements, retention times, precursor ion information, and characteristic MS/MS fragmentation patterns. The obtained data were compared with the MassLynx spectral database and relevant published literature. Compound assignments were based on agreement between precursor ions, major fragment ions, and previously reported phytochemicals from Zygophyllum species and related taxa. Because authentic reference standards were not analyzed under identical chromatographic conditions, all compounds reported in this study should be considered tentatively identified (Metabolomics Standards Initiative, MSI Level 2 confidence).

### 3.15. Separation of ZFL Crude Extract by Different Solvent Systems

The separation of the ZFL crude extract by different solvent systems was determined based on a modified method of Abubakar and Haque [55]. The crude ethanol extract was dissolved in water at room temperature and extracted three times with petroleum ether (1:1) in a separating funnel. The combined petroleum ether layers were concentrated using a rotary evaporator at 35 °C. The aqueous layer was then extracted three times with dichloromethane (1:1) three times and the combined dichloromethane layers were concentrated at 40 °C. Subsequent extractions were performed with ethyl acetate (1:1) and butanol (1:1), each of which repeated three times. The combined extracts were separately concentrated using a rotary evaporator at 45 °C. The remaining aqueous solution was also dried on a rotary evaporator at 50 °C. All dry extracts were weighed.

### 3.16. Microbiological Analysis

For antibacterial activity, *Staphylococcus aureus* ATCC 6538, *Escherichia coli* ATCC 8939, *Enterococcus faecalis* ATCC 29212, and *Pseudomonas aeruginosa* ATCC 9027 were selected. For antifungal activity, *Candida albicans* ATCC 10231, *Aspergillus niger* ATCC 16404, *Malassezia furfur* ATCC 14521, and *Trichophyton rubrum* ATCC 28188 were chosen.

Five fractions of ZFL plant extract, along with model drugs nystatin and tetracycline × HCl, were dissolved in 1 mL of 100% DMSO as a solvent. A portion (0.1 mL) of this mixture was then diluted with 0.9 mL of Mueller–Hinton Broth (MHB) for the antibacterial assay and Potato Dextrose Broth (PDB) for the antifungal assay to achieve the desired sample concentration with 10% DMSO. Tetracycline and nystatin at concentrations 1 mg/mL were used as positive controls.

### 3.17. Preparation of Bacterial and Fungal Suspensions

The preparation of bacterial and fungal suspensions was carried out following a modified method of Julianti et al. [56]. Using an Ose needle, the microbe of interest was streaked onto an agar slant and incubated at 35 ± 2 °C for 24 h for bacteria, and at 25 ± 2 °C for 3–5 days for fungi. The following day, the microbial growth on agar slant surface was removed, suspended in MHB or PDB, and incubated. The suspension was then diluted to reach an absorbance of 0.08–0.13 at 625 nm for bacteria (equivalent to 1 × 10^8^ CFU/mL) and 0.12–0.15 at 530 nm for fungi (equivalent to 1 × 106 CFU/mL). Once the absorbance criteria were met, further dilutions were made to achieve final cell concentrations of 5 × 10^5^ CFU/mL for bacteria and 0.5 × 10^3^ CFU/mL for fungi.

### 3.18. Determination of the Colony Forming Unit

To accurately measure the microbial suspension cell concentration, a total plate count (TPC) for bacteria, and a yeast and mould count (YMC) for fungi were performed. Microbe were grown on Petri dishes by mixing 1 mL of each serial dilution with 15–20 mL of nutrient agar (NA) for bacteria and potato dextrose agar (PDA) for fungi. The plates were then incubated at 35 ± 2 °C for 24 h for bacteria and at 25 ± 2 °C for 3–5 days for fungi. Colony-forming units (CFU) were counted using a colony counter. The concentrations of the initial bacterial and fungal suspensions (stock) were calculated from the Petri dishes containing 30–300 bacterial colonies and 5–50 fungal colonies.

### 3.19. Antibacterial and Antifungal Screening

Antibacterial and antifungal screenings were performed using the disc diffusion method according to a modified procedure described by Kumari and Menghani [57]. All experiments were conducted in triplicate.

Briefly, 20 mL of Mueller–Hinton agar (MHA) for bacterial strains or potato dextrose agar (PDA) for fungal strains was poured into sterile Petri dishes. After solidification, 1 mL of microbial suspension (1 × 10^8^ CFU/mL) was uniformly spread on the agar surface. Sterile paper discs (6 mm diameter) were impregnated with 10 μL of the corresponding ZFL fraction (petroleum ether, dichloromethane, ethyl acetate, butanol, or water fraction) and placed onto the inoculated agar plates. Tetracycline × HCl and nystatin were used as positive controls for bacteria and fungi, respectively. The extracts were dissolved in 10% DMSO, which was also used as the negative control.

The plates were incubated at 35 ± 2 °C for 24 h for bacteria and at 25 ± 2 °C for 3–5 days for fungi. After incubation, inhibition-zone diameters were measured and expressed as mean ± SD (*n* = 3 independent experiments).

The reported inhibition-zone diameters include the diameter of the paper disc (6 mm) and are expressed as mean ± standard deviation (SD) of three independent experiments. No inferential statistical analysis was performed; therefore, the antimicrobial results should be considered preliminary screening data intended to evaluate the susceptibility of the tested microorganisms to the different solvent fractions.

### 3.20. Determination of the Minimum Inhibitory Concentration (MIC)

The Minimum Inhibitory Concentration (MIC) was determined using a modified method of Eloff [22]. The MIC values of the ZFL herb extracts against bacteria and fungi were assessed with the microdilution method in a 96-well microplate. Initially, 196 µL of microbial suspension (final concentration of 5 × 10^5^ CFU/mL for bacteria and 0.5 × 10^3^ CFU/mL for fungi) was added to the first column, and 100 µL was added to each subsequent column. Next, 4 µL of samples (starting at a concentration of 50 mg/mL) were added to the first column (final volume of 200 µL, final concentration of 1000 µg/mL), homogenized, and then 100 µL was transferred from the first column to the second column, continuing until the final dilution was achieved. The excess solution in the last column was discarded. The same steps were applied for nystatin and tetracycline HCl as positive controls. The plates were incubated at 35 ± 2 °C for 18–24 h for bacteria and at 25 ± 2 °C for 3 days for fungi.

### 3.21. Cytotoxic Assay on A. salina

The cytotoxic activity of the ethanol extract of ZFL was investigated using a survival test on *Artemia salina* crustaceans. For the experiment, flasks were filled with artificial seawater, into which *A. salina eggs* were added. Incubation was carried out for three days at a temperature of 20 ± 5 °C and a natural photoperiod, ensuring gentle aeration, until the nauplii hatched. The salinity of the control water was maintained at 8.0–8.5 pH.

Solutions with concentrations of 10, 5 and 1 mg/mL were prepared from the ethanol extract of the plant. Twenty- to forty-one-day-old larvae per test tube were used for testing. The larvae were exposed to the specified concentrations in separate series of experiments. Actinomycin D was used as a control drug. The mortality rate was determined by the ratio of the number of dead larvae to their total number.

The assay was performed in triplicate, and mortality was recorded and expressed as mean ± SD (*n* = 3 independent experiments). Cytotoxicity was expressed as percentage mortality relative to the untreated control group.

MBC and MFC values were not determined in the present study; therefore, the antimicrobial results are interpreted based on inhibition-zone diameters and MIC values only. Consequently, no bactericidal or fungicidal conclusions are drawn, and further studies are required to determine the MBC/MFC values of the active fractions.

## 4. Conclusions

The pharmacognostic characteristics of ZFL growing in the Zhetysu region of Kazakhstan were investigated to generate baseline scientific data relevant to the characterization and quality assessment of this medicinal plant. Microscopic analysis of the aerial parts allowed for reliable identification of the plant raw material and revealed anatomical features typical of xeromorphic and succulent plants.

Phytochemical analysis confirmed that ZFL contains a diverse range of secondary metabolites, including alkanes, alcohols, terpenes, carboxylic acids, and phenyl derivatives. Quantitative analyses also demonstrated the presence of various biologically important compounds such as flavonoids, coumarins, alkaloids, saponins, organic acids, and polysaccharides, as well as essential mineral elements.

Biological evaluation demonstrated that several ZFL fractions exhibited measurable antibacterial and antifungal activity against selected pathogenic microorganisms. However, these findings should be considered preliminary and require further confirmation through additional microbiological investigations and bioassay-guided studies.

Overall, the obtained results provide comprehensive pharmacognostic, phytochemical, and preliminary biological information on ZFL populations growing in Kazakhstan and may contribute to future quality-control and standardization studies. Further investigations should include the isolation and characterization of active constituents, elucidation of their mechanisms of action, evaluation of additional plant populations, and in vivo pharmacological and safety studies.

## Figures and Tables

**Table 1:** GC-MS analysis results.

Entry	Compounds	Molecular Formula	Molecular Mass (g/mol)	PubChem CID	Similarity	Area (%)
1	Octacosane	C_28_H_58_	394.8	12,408	92	13.7
2	Hexadecanoic acid	C_16_H_32_O_2_	256.42	985	93	8.22
3	Octadecane, 3-ethyl-5-(2-ethylbutyl)-	C_26_H_54_	366.7	292,285	70	7.28
4	Behenic alcohol	C_22_H_46_O	326.6	12,620	92	5.70
5	Octacosanol	C_28_H_58_O	410.8	68,406	93	4.23
6	E-15-Heptadecenal	C_17_H_32_O	252.4	5,363,097	94	4.06
7	Tetracosanol-1	C_24_H_50_O	354.7	10,472	92	3.50
8	9,12,15-Octadecatrienoic acid (Z,Z,Z)	C_18_H_30_O_2_	278.4	5,280,934	91	3.38
9	Phytol	C_20_H_40_O	296.5	5,280,435	94	2.98
10	12-Tricosanone	C_23_H_46_O	338.6	10,888	70	2.57
11	1-Decanol, 2-hexyl-	C_16_H_34_O	242.44	95,337	82	2.04
12	1-Nonene, 4,6,8-trimethyl-	C_12_H_24_	168.32	41,077	78	2.01
13	Squalene	C_30_H_50_	410.7	638,072	96	1.88
14	1-Decanol, 2-octyl-	C_18_H_38_O	270.5	3,084,890	80	1.81
15	1-Dodecanol, 2-octyl-	C_20_H_42_O	298.5	21,414	78	1.77
16	Heneicosane	C_21_H_44_	296.6	12,403	84	1.76
17	1-Dodecanol, 2-hexyl-	C_18_H_38_O	270.5	86,112	81	1.67
18	Hexadecane, 2,6,11,15-tetramethyl-	C_20_H_42_	282.5	136,331	85	1.36
19	Benzoic acid, 4-ethoxy-, ethyl ester	C_11_H_14_O_3_	194.23	90,232	71	1.29
20	1-Nonadecene	C_19_H_38_	266.5	29,075	94	1.25
21	Heptadecane	C_17_H_36_	240.5	12,398	84	1.24
22	Hexacosane	C_26_H_54_	366.7	12,407	88	1.19
23	Hexacosane, 9-octyl-	C_34_H_70_	478.9	296,567	85	1.19
24	Octadecanal	C_18_H_36_O	268.5	12,533	90	1.01
25	Dibutyl phthalate	C_16_H_22_O_4_	278.34	3026	92	0.87
26	Nonadecane	C_19_H_40_	268.5	12,401	90	0.82
27	Triacontanoic acid, methyl ester	C_31_H_62_O_2_	466.8	12,400	68	0.82
28	Butylated hydroxytoluene	C_15_H_24_O	220.35	31,404	87	0.80
29	Hexadecane	C_16_H_34_	226.44	11,006	95	0.74
30	Dodecane, 4,6-dimethyl-	C_14_H_30_	198.39	545,627	86	0.68
31	4,8,12,16-Tetramethylheptadecan-4-olide	C_21_H_40_O_2_	324.5	567,149	90	0.67
32	Octacosanoic acid methyl ester	C_29_H_58_O_2_	438.8	41,518	84	0.61
33	Octadecane	C_18_H_38_	254.5	11,635	94	0.55
34	Hexacosanoic acid, methyl ester	C_27_H_54_O_2_	410.7	22,048	81	0.52
35	Pentadecane	C_15_H_32_	212.41	12,391	92	0.50
36	9,12,15-Octadecatrienoic acid methyl ester	C_19_H_32_O_2_	292.5	5,319,706	83	0.50
37	Cetene	C_16_H_32_	224.42	12,395	90	0.49
38	Oxirane, hexadecyl	C_18_H_36_O	268.5	23,872	79	0.45
39	Octadecanoic acid	C_18_H_36_O_2_	284.5	5281	83	0.44
40	Hexadecane, 2,6,10,14-tetramethyl-	C_20_H_42_	282.5	12,523	79	0.43
41	(E)-β-Farnesene	C_15_H_24_	204.35	10,407	86	0.42
42	Eicosane, 2-methyl-	C_21_H_44_	296.6	519,146	83	0.42
43	9,12-Octadecadienoic acid methyl ester	C_19_H_34_O_2_	294.5	5,284,421	84	0.40
44	Pentadecane, 2,6,10,14-tetramethyl-	C_19_H_40_	268.5	15,979	83	0.33
45	Phenol, 2,4-bis(1,1-dimethylethyl)	C_14_H_22_O	206.32	7311	90	0.30
46	8-Amino-6-methoxy-5-[n-propoxy]quinoline	C_13_H_16_N_2_O_2_	232.28	605,075	68	0.30
47	Heptadecane, 9-octyl-	C_25_H_52_	352.7	292,286	75	0.29
48	2′,4′-Dimethoxy-3′-methylpropiophenone	C_12_H_16_O_3_	208.25	601,578	70	0.27
49	Hentriacontane	C_31_H_64_	436.8	12,410	76	0.27
50	Isopropyl myristate	C_17_H_34_O_2_	270.5	8042	83	0.18
51	Oleic acid	C_18_H_34_O_2_	282.5	445,639	81	0.09

**Table 2:** Tentatively identified compounds detected by LC-MS analysis.

№	RT (min)	Area %	Formula	Compound Name	Precursor Ion (*m*/*z*)	Fragment Ions (*m*/*z*)	Structure	Reference
1	10.899	28.63	C_16_H_12_O_5_	Acacetin	285.1462 [M+H]^+^	270, 242, 151	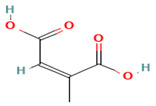	El-Haddad et al., 2025 [23]; Hamed et al., 2024 [24]
2	16.833	14.65	C_29_H_50_O	β-Sitosterol	415.3119 [M+H]^+^	397, 381, 303	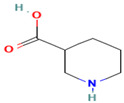	Hamid et al., 2016 [14]; Hamed et al., 2024 [24]
3	18.26	7.12	C_30_H_48_O_3_	Oleanolic acid	457.3742 [M+H]^+^	439, 411, 203	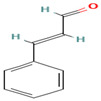	Singh et al., 2025 [25]; Hamed et al., 2024 [24]
4	6.027	4.69	C_15_H_10_O_7_	Quercetin	303.0520 [M+H]^+^	285, 257, 229	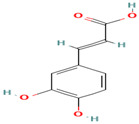	El-Haddad et al., 2025 [23]; Hamed et al., 2024 [24]
5	3.257	4.33	C_9_H_8_O	Cinnamaldehyde	132.3512 [M+H]^+^	103, 77	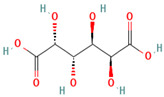	El-Haddad et al., 2025 [23]
6	17.958	4.32	C_30_H_50_O	β-Amyrin	427.3749 [M+H]^+^	409, 218, 203	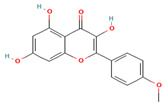	Hamed et al., 2024 [24]
7	6.638	4.29	C_15_H_10_O_6_	Kaempferol	287.0569 [M+H]^+^	269, 241, 153	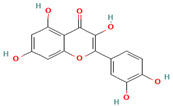	Hamid et al., 2016 [14]; Hamed et al., 2024 [24]
8	1.324	3.98	C_5_H_6_O_4_	Citraconic acid	131.0149 [M+H]^+^	113, 85	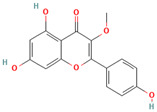	El-Haddad et al., 2025 [23]
9	2.793	3.15	C_6_H_11_NO_2_	Nipecotic acid	130.0624 [M+H]^+^	84, 56	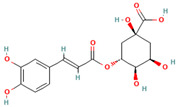	Singh et al., 2025 [25]
10	12.551	3.02	C_11_H_12_O_5_	Sinapinic acid	225.2029 [M+H]^+^	207, 175, 147	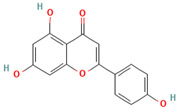	Singh et al., 2025 [25]
11	9.936	2.88	C_15_H_10_O_5_	Apigenin	271.1300 [M+H]^+^	253, 225, 151	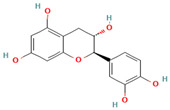	El-Haddad et al., 2025 [23]
12	7.061	2.43	C_16_H_18_O_9_	Chlorogenic acid	355.1709 [M+H]^+^	191, 179, 135	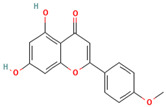	El-Haddad et al., 2025 [23]
13	10.463	2.25	C_15_H_14_O_6_	Catechin	291.1456 [M+H]^+^	273, 245, 205	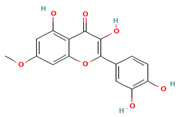	Hamed et al., 2024 [24]
14	12.242	2.24	C_16_H_12_O_7_	Isorhamnetin	317.1614 [M+H]^+^	302, 287, 153	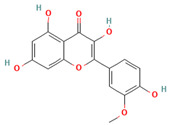	Hamid et al., 2016 [14]
15	13.163	2.00	C_9_H_8_O_7_S	Caffeic acid-3-sulfate	261.2238 [M+H]^+^	179, 135	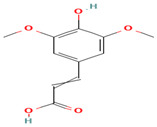	Singh et al., 2025 [25]
16	11.777	1.87	C_16_H_12_O_7_	Rhamnetin	317.1924 [M+H]^+^	302, 287	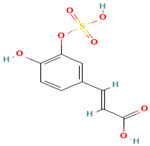	Hamed et al., 2024 [24]
17	13.627	1.77	C_20_H_20_O_6_	Coniferyl ferulate	357.2376 [M+H]^+^	339, 177	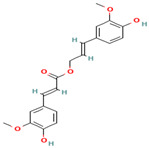	Singh et al., 2025 [25]
18	14.091	1.16	C_14_H_21_NO_3_	Furmetamide	252.2347 [M+H]^+^	207, 179	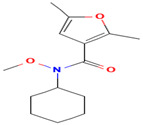	Singh et al., 2025 [25]
19	5.015	1.07	C_6_H_10_O_8_	Mucate (galactari c acid)	211.1557 [M+H]^+^	193, 147	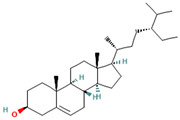	El-Haddad et al., 2025 [23]
20	4.747	0.92	C_9_H_8_O_4_	Caffeic acid	181.0760 [M+H]^+^	163, 135	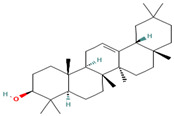	El-Haddad et al., 2025 [23]
21	5.366	0.86	C_16_H_12_O_6_	Kaempferide	301.0519 [M+H]^+^	286, 258	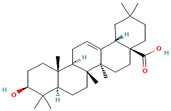	Hamed et al., 2024 [24]

**Table 3:** Quantitative analyses of ZFL.

	Quantitative Analyses	Content (%)
1	Extractive substances	15.419
2	Flavonoids in terms of quercetin	0.084
3	Alkaloids	0.654
4	Tannins	5.574
5	Saponins	0.306
6	Polysaccharides	1.564
7	Coumarins	2.775
8	Organic acids	0.225

**Table 4:** Macro and microelements contained in ZFL.

Element	Content (mg/kg DW)
Cu	0.2080
Fe	2.2260
Zn	0.5120
Ni	0.1264
Mn	0.6317
Pb	Not detected
Cd	0.0178
Ca	520.3120
Mg	93.9839
K	601.9088
Na	362.6227

**Table 5:** ZFL fraction weights.

ZFL Extract Solvents	Weight (g)
Petroleum ether	0.241
Dichloromethane	0.437
Ethyl acetate	0.092
Butanol	0.630
Water	1.425

**Table 6:** Results of Assessment of Inhibitory Zone Diameter Measurement against Bacteria. Representative photographs of the antibacterial disc diffusion assay are provided in Supplementary Figure S22. The figure illustrates the antibacterial activity of the ZFL fractions against selected bacterial strains, including *E. faecalis*, *S. aureus*, and *E. coli*. The sample order on each plate is indicated in the figure caption, allowing direct comparison of the inhibition zones produced by the different solvent fractions.

Name of Sample	*E. faecalis*, mm	*S. aureus*, mm	*E. coli*, mm	*P. aeruginosa*, mm
Positive control (tetracycline)	7 ± 1.2	11 ± 1.1	7.6 ± 1.2	12.2 ± 2.3
Negative control (DMSO)	0 ± 0.0 mm	6 ± 1.2	0 ± 0.0 mm	0 ± 0.0 mm
Ethyl acetate fraction	7 ± 1.2	7.6 ± 1.2	6.5 ± 1.2	9 ± 1.2
Petroleum ether fraction	10 ± 1.2	8.4 ± 1.2	7.25 ± 1.2	0 ± 0.0 mm
Dichloromethane fraction	8 ± 1.5	11.6 ± 1.1	8.3 ± 1.2	0 ± 0.0 mm
Butanol fraction	9 ± 1.2	9.2 ± 1.2	10 ± 1.1	10.3 ± 1.2
Water fraction	0 ± 0.0 mm	10.2 ± 1.2	8.8 ± 1.2	11.3 ± 1.1

Values are expressed as mean ± SD (*n* = 3 independent experiments).

**Table 7:** Results of Assessment of Inhibitory Zone Diameter Measurement for Fungi. Representative photographs of the antifungal disc diffusion assay are provided in Supplementary Figure S23. The figure illustrates the antifungal activity of the ZFL fractions against *C. albicans*, *M. furfur*, and *A. niger*. The sample order on each plate and the control groups are indicated in the figure caption.

Name of Sample	*C. albicans*, mm	*M. furfur*, mm	*T. rubrum*, mm	*A. niger*, mm
Positive control (Nystatin)	20 ± 1.1	19.3 ± 1.1	20 ± 1.1	20 ± 1.1
Negative control (DMSO)	4.6 ± 1.3	4.6 ± 1.3	0 ± 0.0 mm	0 ± 0.0 mm
Ethyl acetate fraction	7.3 ± 1.2	9.9 ± 1.1	0 ± 0.0 mm	9 ± 1.2
Petroleum ether fraction	11 ± 1.2	13.6 ± 1.1	0 ± 0.0 mm	0 ± 0.0 mm
Dichloromethane fraction	9.3 ± 1.2	16 ± 1.1	0 ± 0.0 mm	8.6 ± 1.2
Butanol fraction	14.3 ± 1.1	14 ± 1.1	0 ± 0.0 mm	9.6 ± 1.2
Water fraction	10.6 ± 1.1	10.3 ± 1.1	0 ± 0.0 mm	0 ± 0.0 mm

Values are expressed as mean ± SD (*n* = 3 independent experiments).

**Table 8:** MIC of the fractions against the tested microbes.

Minimum Inhibitory Concentration (MIC) (µg/mL)								
Fractions	*E. faecalis*	*S. aureus*	*E.coli*	*P. aeruginosa*	*C. albicans*	*M. furfur*	*T. rubrum*	*A. niger*
Ethyl acetate	500	1000	>1000	250	>1000	>1000	>1000	>1000
Petroleum ether	>1000	>1000	>1000	>1000	>1000	>1000	>1000	>1000
Dichloromethane	>1000	>1000	>1000	>1000	>1000	>1000	>1000	>1000
Butanol	>1000	>1000	>1000	>1000	1000	250	>1000	250
Water	>1000	>1000	>1000	500	>1000	>1000	>1000	>1000
Tetracycline	12.5	12.5	12.5	12.5	NI	NI	NI	NI
Nystatin	NI	NI	NI	NI	100	50	>100	50

NI = no inhibition.

**Table 9:** Results of the cytotoxic activity study against *Artemia salina*.

Treatments	Concentrations (mg/mL)	% of Surviving Larvae	Mortality (%)
Actinomycin D	1	22	74
	5	2	94
	10	0	96
Ethanolic extract	1	96	0
	5	96	0
	10	96	0

## Data Availability

The original contributions presented in this study are included in the article/Supplementary Material. Further inquiries can be directed to the corresponding authors.

## Supplementary Materials

The following supporting information can be downloaded at https://www.mdpi.com/article/10.3390/ijms27135907/s1 [57,58,59,60,61,62,63,64,65,66,67,68,69,70,71,72,73,74,75,76,77,78,79,80,81,82,83].

## Abbreviations

The following abbreviations are used in this manuscript:

ZFL	*Zygophyllum fabago* L.
MIC	Minimum Inhibitory Concentration
GC-MS	Gas chromatography–mass spectrometry
DMSO	Dimethyl sulfoxide
MHA	Mueller–Hinton agar
PDA	Potato dextrose agar
MHB	Mueller–Hinton Broth
PDB	Potato Dextrose Broth
