# Supplementary material for: Pharmacognostic Characterization, Phytochemical Profiling, and In Vitro Biological Evaluation of Zygophyllum fabago L
Source: Int J Mol Sci. 2026 Jun 30;27(13):5907. doi: 10.3390/ijms27135907 (PMC13361426; doi:10.3390/ijms27135907)
Supplement: Supplementary file 1 [file ijms-27-05907-s001.zip › Figures S1–S21. Mass spectra of tentatively identified compounds in the 70% ethanolic extract of Zygophyllum fabago L..pdf]

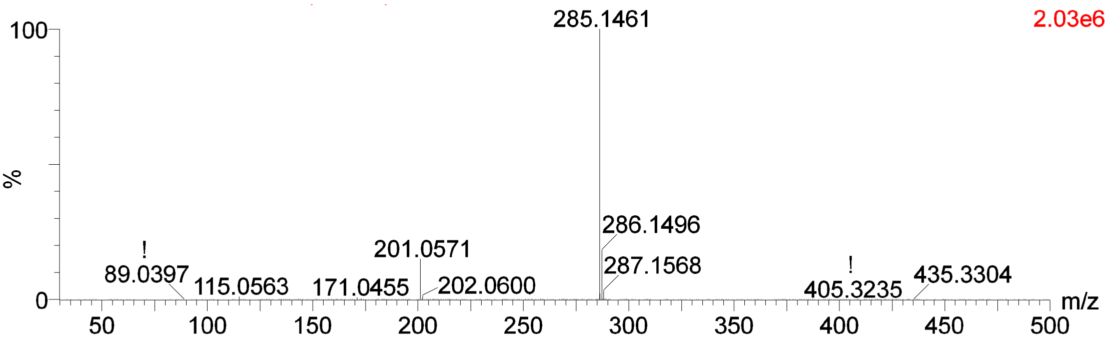

Figure S1. Mass spectrum of acacetin tentatively identified in the 70% ethanolic extract of *Zygophyllum fabago* L.; RT = 10.899 min; precursor ion: m/z 285.1462 [M+H]+; major fragment ions: m/z 270, 242, and 151.

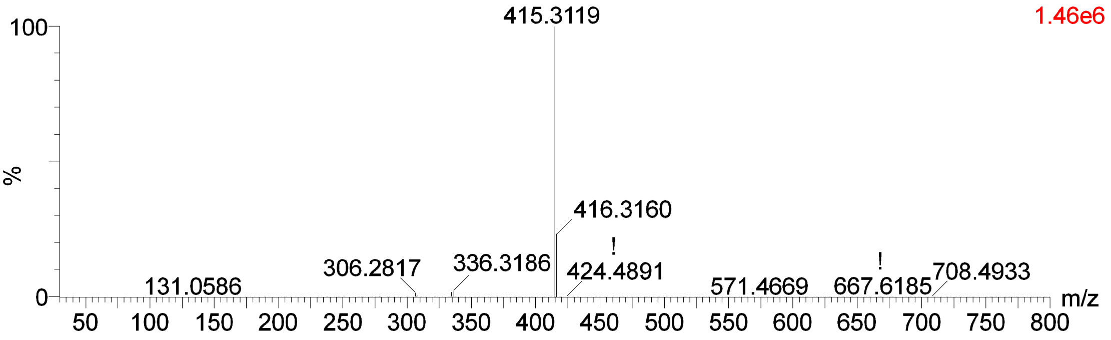

Figure S2. Mass spectrum of β-sitosterol tentatively identified in the 70% ethanolic extract of *Zygophyllum fabago* L.; RT = 16.833 min; precursor ion: m/z 415.3119 [M+H]+; major fragment ions: m/z 397, 381, and 303.

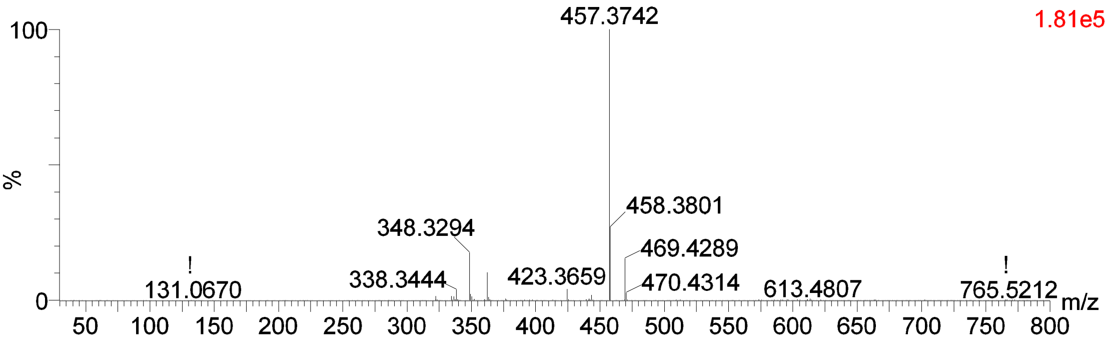

Figure S3. Mass spectrum of oleanolic acid tentatively identified in the 70% ethanolic extract of *Zygophyllum fabago* L.; RT = 18.260 min; precursor ion: m/z 457.3742 [M+H]+; major fragment ions: m/z 439, 411, and 203.

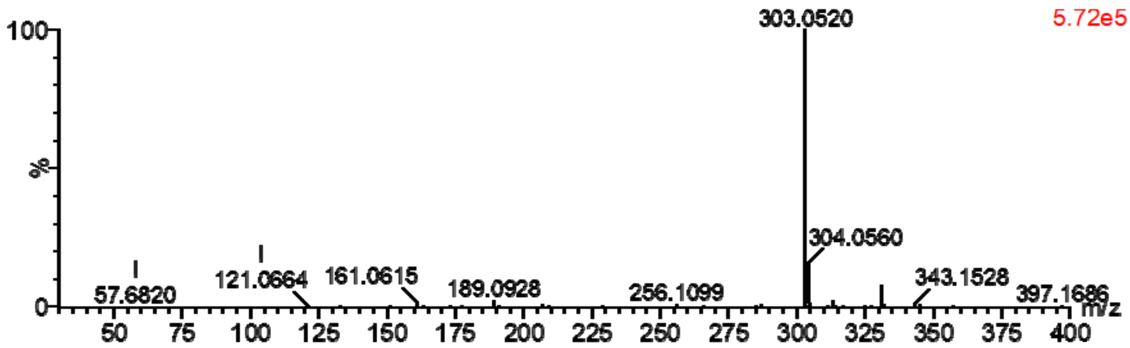

Figure S4. Mass spectrum of quercetin tentatively identified in the 70% ethanolic extract of *Zygophyllum fabago* L.; RT = 6.027 min; precursor ion: m/z 303.0520 [M+H]+; major fragment ions: m/z 285, 257, and 229.

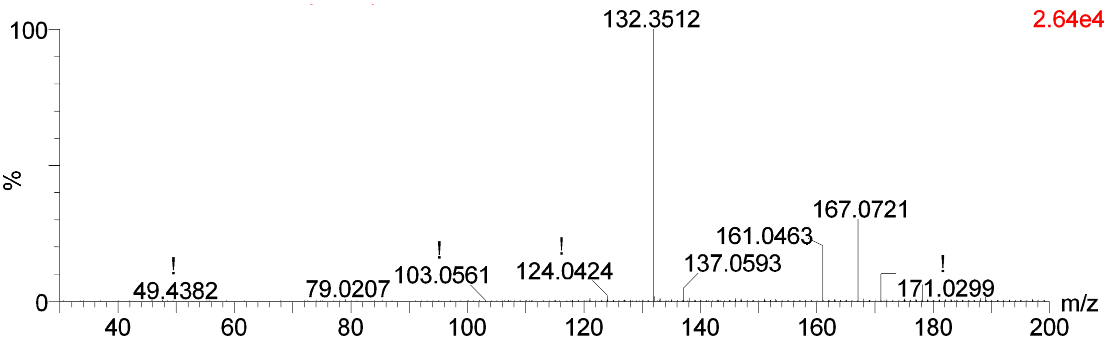

Figure S5. Mass spectrum of cinnamaldehyde tentatively identified in the 70% ethanolic extract of *Zygophyllum fabago* L.; RT = 3.257 min; precursor ion: m/z 132.3512 [M+H]+; major fragment ions: m/z 103 and 77.

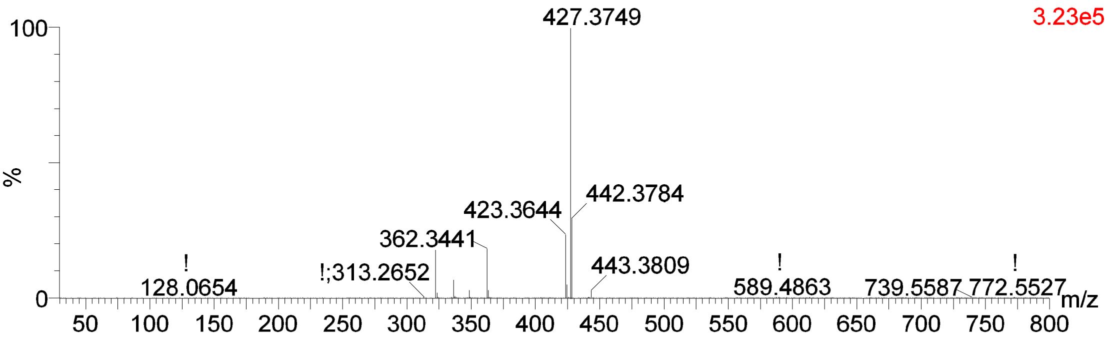

Figure S6. Mass spectrum of β-amyrin tentatively identified in the 70% ethanolic extract of *Zygophyllum fabago* L.; RT = 17.958 min; precursor ion: m/z 427.3744 [M+H]+; major fragment ions: m/z 409, 218, and 203.

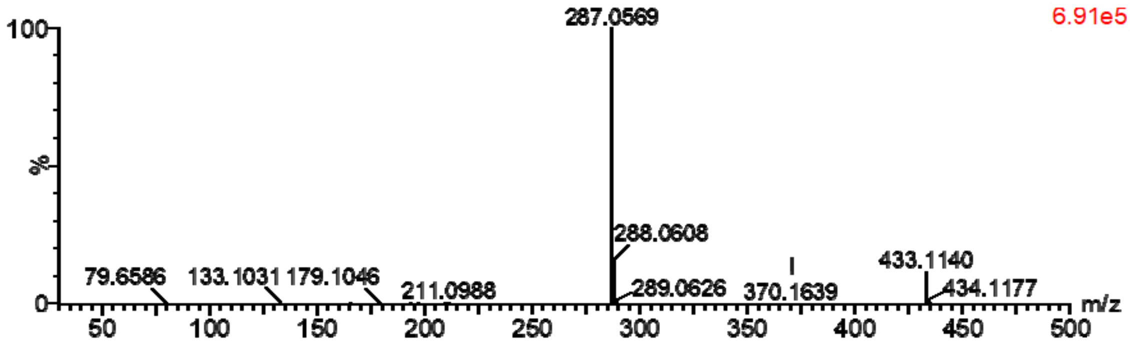

Figure S7. Mass spectrum of kaempferol tentatively identified in the 70% ethanolic extract of *Zygophyllum fabago* L.; RT = 6.638 min; precursor ion: m/z 287.0569 [M+H]+; major fragment ions: m/z 269, 241, and 153.

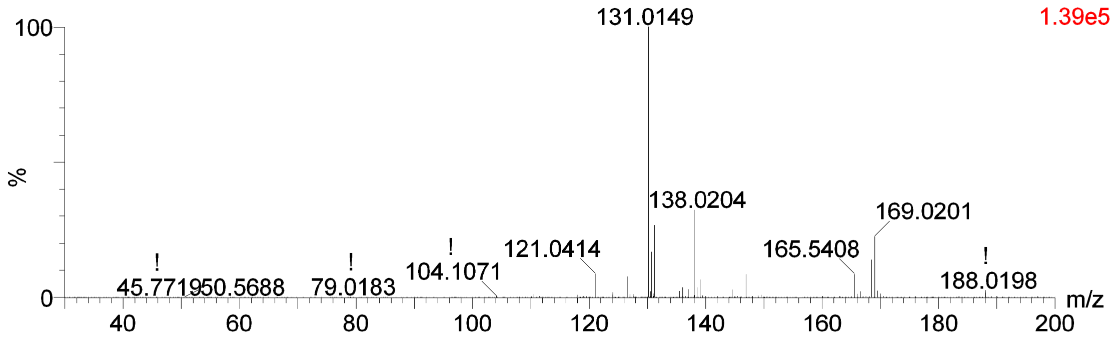

Figure S8. Mass spectrum of citraconic acid tentatively identified in the 70% ethanolic extract of Zygophyllum fabago L.; RT = 1.324 min; precursor ion: m/z 131.0149 [M+H]+; major fragment ions: m/z 113 and 85.

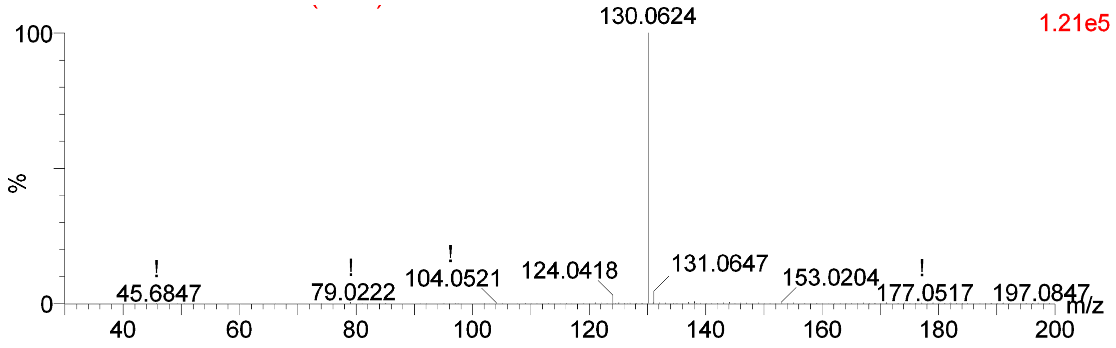

Figure S9. Mass spectrum of nipecotic acid tentatively identified in the 70% ethanolic extract of *Zygophyllum fabago* L.; RT = 2.793 min; precursor ion: m/z 130.0624 [M+H]+; major fragment ions: m/z 84 and 56.

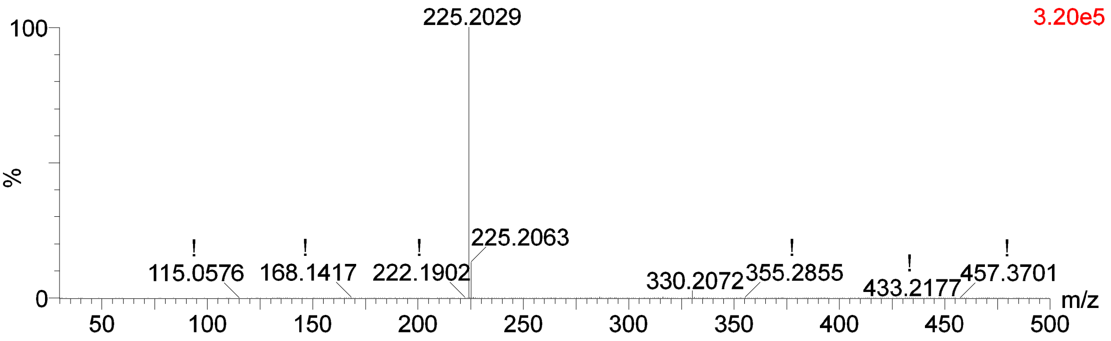

Figure S10. Mass spectrum of sinapinic acid tentatively identified in the 70% ethanolic extract of *Zygophyllum fabago* L.; RT = 12.551 min; precursor ion: m/z 225.2029 [M+H]$^{+}$; major fragment ions: m/z 207, 175, and 147.

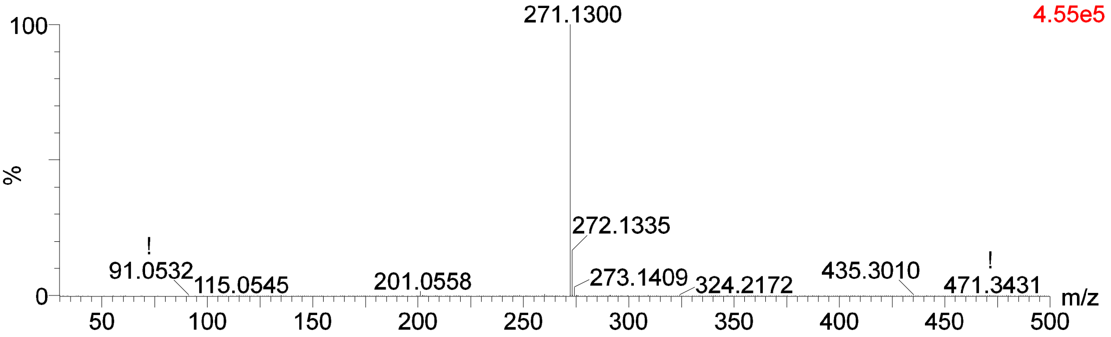

Figure S11. Mass spectrum of apigenin tentatively identified in the 70% ethanolic extract of *Zygophyllum fabago* L.; RT = 9.936 min; precursor ion: m/z 271.1300 [M+H]$^{+}$; major fragment ions: m/z 253, 225, and 151.

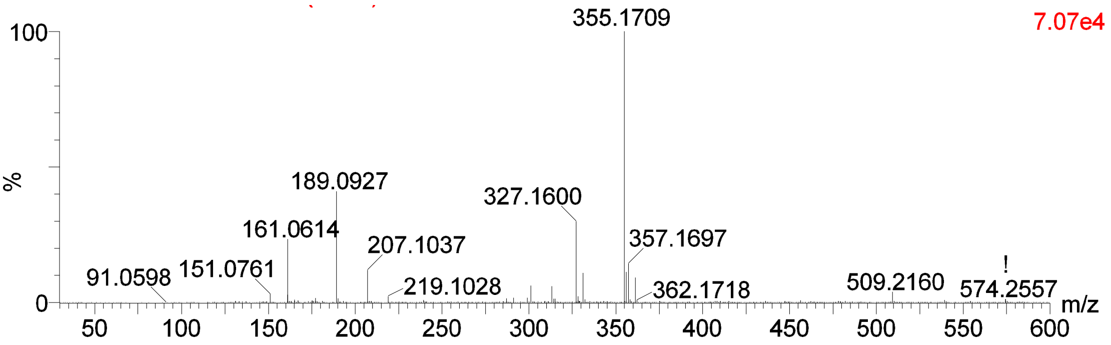

Figure S12. Mass spectrum of chlorogenic acid tentatively identified in the 70% ethanolic extract of *Zygophyllum fabago* L.; RT = 7.061 min; precursor ion: m/z 355.1709 [M+H]$^{+}$; major fragment ions: m/z 191, 179, and 135.

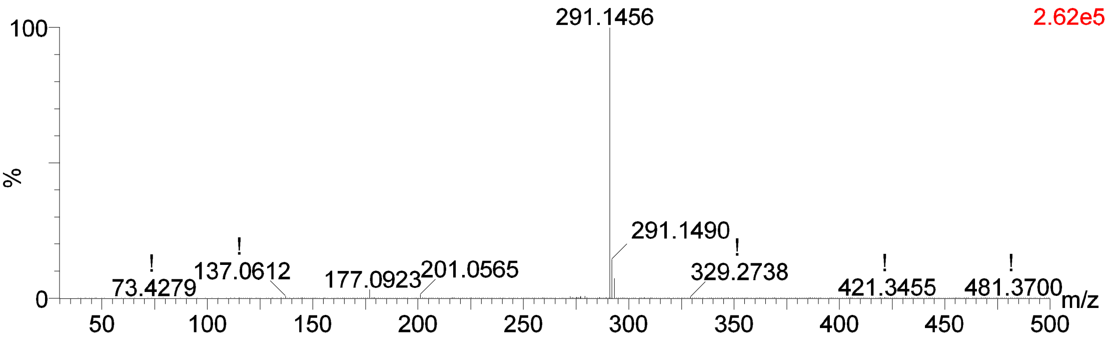

Figure S13. Mass spectrum of catechin tentatively identified in the 70% ethanolic extract of *Zygophyllum fabago* L.; RT = 10.463 min; precursor ion: m/z 291.1456 [M+H]$^+$; major fragment ions: m/z 273, 245, and 205.

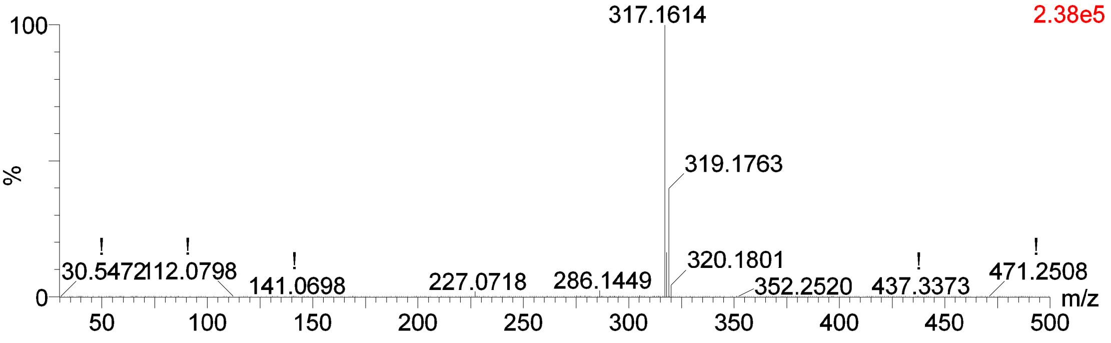

Figure S14. Mass spectrum of isorhamnetin tentatively identified in the 70% ethanolic extract of *Zygophyllum fabago* L.; RT = 12.242 min; precursor ion: m/z 317.1615 [M+H]$^+$; major fragment ions: m/z 302, 287, and 153.

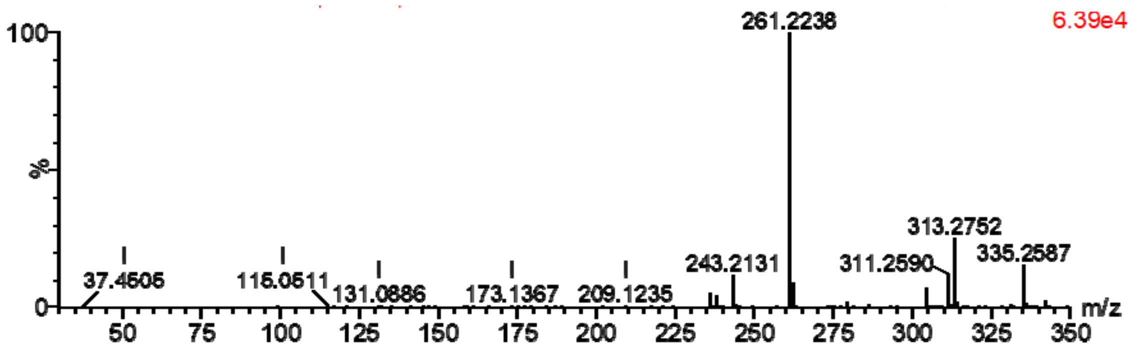

Figure S15. Mass spectrum of caffeic acid-3-sulfate tentatively identified in the 70% ethanolic extract of *Zygophyllum fabago* L.; RT = 13.163 min; precursor ion: m/z 261.2238 [M+H]$^+$; major fragment ions: m/z 179 and 135.

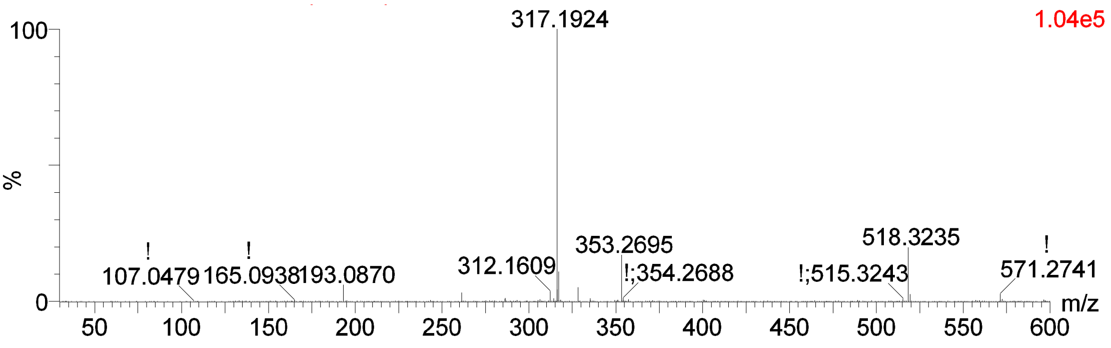

Figure S16. Mass spectrum of rhamnetin tentatively identified in the 70% ethanolic extract of *Zygophyllum fabago* L.; RT = 11.777 min; precursor ion: m/z 317.1924 [M+H]$^+$; major fragment ions: m/z 302 and 287.

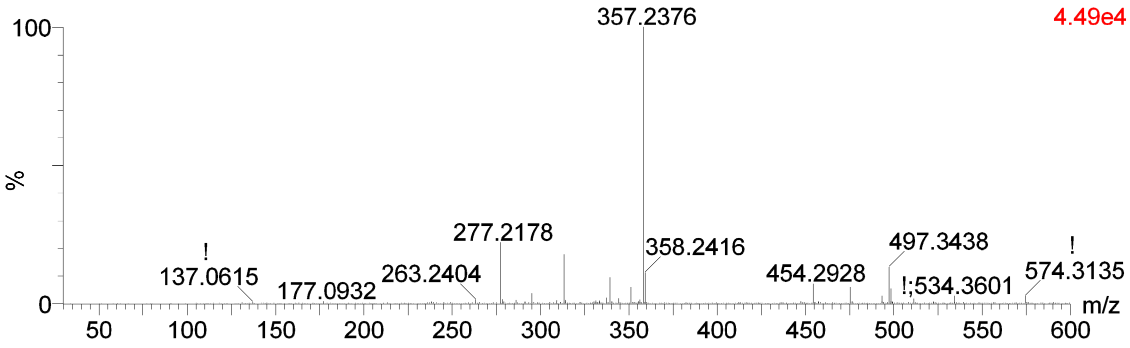

Figure S17. Mass spectrum of coniferyl ferulate tentatively identified in the 70% ethanolic extract of *Zygophyllum fabago* L.; RT = 13.627 min; precursor ion: m/z 357.2376 [M+H]$^+$; major fragment ions: m/z 339 and 177.

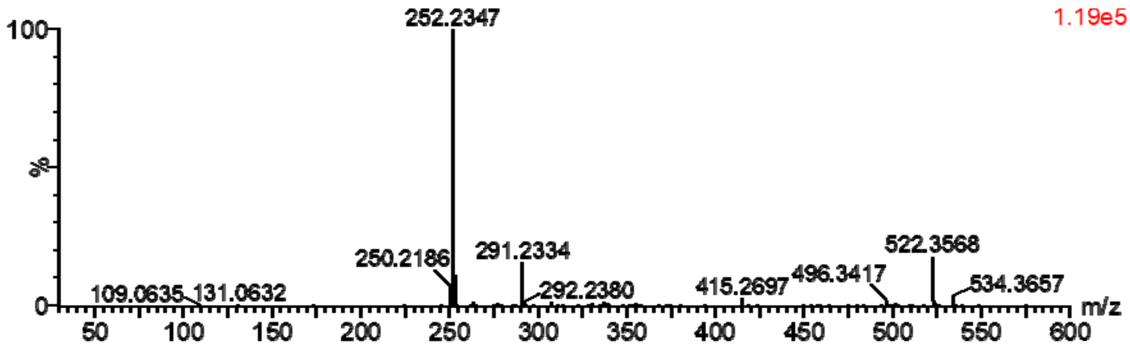

Figure S18. Mass spectrum of furmetamide tentatively identified in the 70% ethanolic extract of *Zygophyllum fabago* L.; RT = 14.091 min; precursor ion: m/z 252.2347 [M+H]$^+$; major fragment ions: m/z 207 and 179.

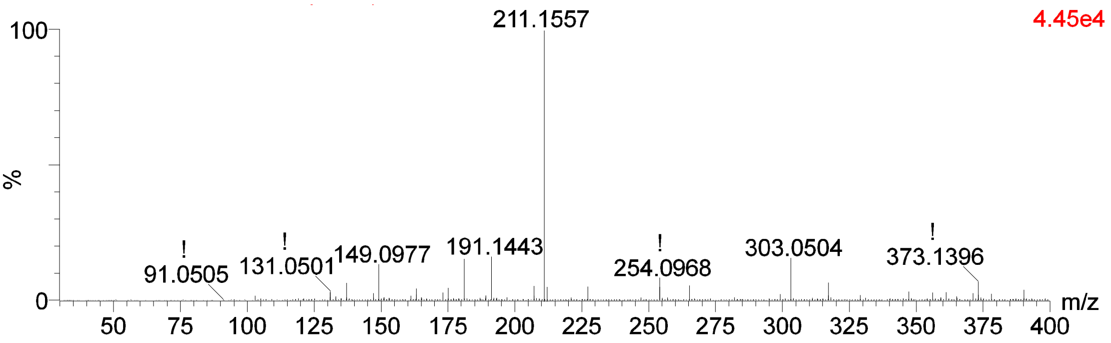

Figure S19. Mass spectrum of mucate (galactaric acid) tentatively identified in the 70% ethanolic extract of *Zygophyllum fabago* L.; RT = 5.015 min; precursor ion: m/z 211.1557 [M+H]$^+$; major fragment ions: m/z 193 and 147.

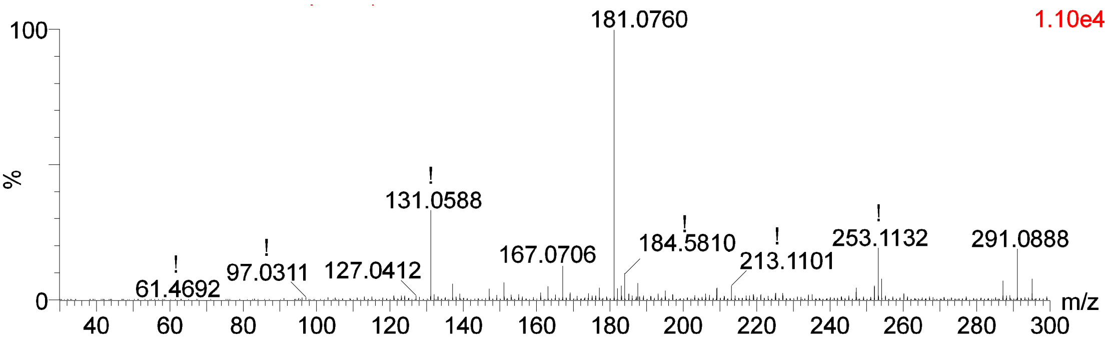

Figure S20. Mass spectrum of caffeic acid tentatively identified in the 70% ethanolic extract of *Zygophyllum fabago* L.; RT = 4.747 min; precursor ion: m/z 181.0760 [M+H]$^+$; major fragment ions: m/z 163 and 135.

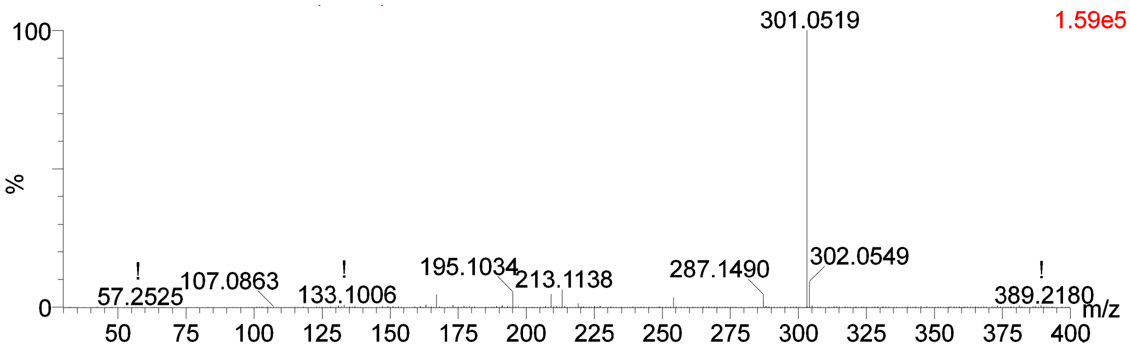

Figure S21. Mass spectrum of kaempferide tentatively identified in the 70% ethanolic extract of *Zygophyllum fabago* L.; RT = 5.366 min; precursor ion: m/z 301.0519 [M+H]$^+$; major fragment ions: m/z 286 and 258.

Figures S1-21. Mass Spectrum of ZFL Extract
