# Supplementary material for: Pharmacognostic Characterization, Phytochemical Profiling, and In Vitro Biological Evaluation of Zygophyllum fabago L
Source: Int J Mol Sci. 2026 Jun 30;27(13):5907. doi: 10.3390/ijms27135907 (PMC13361426; doi:10.3390/ijms27135907)
Supplement: Supplementary file 1 [file ijms-27-05907-s001.zip › Supplementary Figures S22–S23. Representative Antimicrobial Disc Diffusion Assay Photographs of ZFL Fractions.pdf]

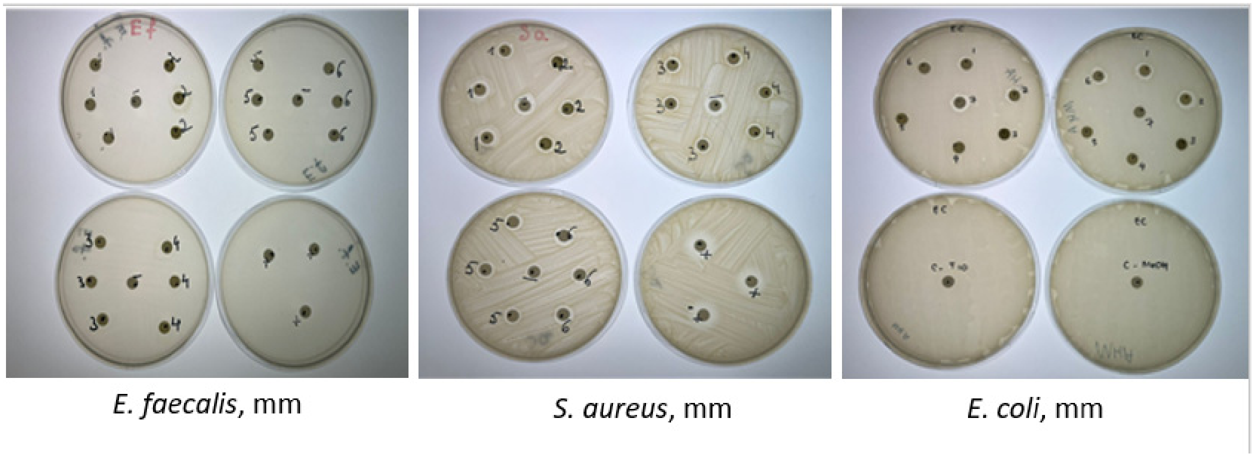

Figure S22. Representative photographs of the antibacterial activity of *Zygophyllum fabago* L. fractions against selected bacterial strains (*Enterococcus faecalis, Staphylococcus aureus, and Escherichia coli*) in the disc diffusion assay. Sample order in each plate was as follows: 1 - ethyl acetate fraction, 2 - petroleum ether fraction, 3 - dichloromethane fraction, 4 - butanol fraction, and 5 - water fraction. C+ denotes the positive control (tetracycline), and C− denotes the negative control (DMSO). Inhibition zones were measured in mm.

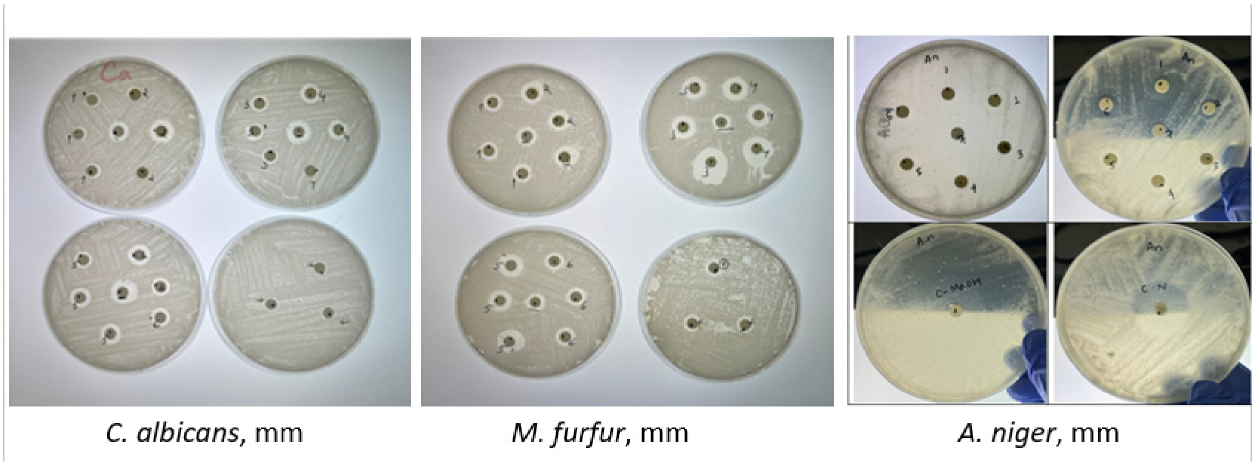

Figure S23. Representative photographs of the antifungal activity of *Zygophyllum fabago* L. fractions against *Candida albicans, Malassezia furfur, and Aspergillus niger* in the disc diffusion assay. Sample order in each plate was as follows: 1 - ethyl acetate fraction, 2 - petroleum ether fraction, 3 - dichloromethane fraction, 4 - butanol fraction, and 5 - water fraction. C+ denotes the positive control (nystatin), and C− denotes the negative control (DMSO). Inhibition zones were measured in mm.
