# Supplementary material for: Pharmacognostic Characterization, Phytochemical Profiling, and In Vitro Biological Evaluation of Zygophyllum fabago L
Source: Int J Mol Sci. 2026 Jun 30;27(13):5907. doi: 10.3390/ijms27135907 (PMC13361426; doi:10.3390/ijms27135907)
Supplement: Supplementary file 1 [file ijms-27-05907-s001.zip › Table S1. Chemical classes and reported biological activities of compounds tentatively identified in the 70% ethanolic extract of Zygophyllum fabago L. by GC-MS analysis..pdf]

Table S1. Chemical classes and reported biological activities of compounds tentatively identified in the 70% ethanolic extract of *Zygophyllum fabago* L. by GC-MS analysis.

GC-MS entry No.	Chemical class	Compound	Reported biological activity in the literature	Ref.
1	Alkanes	Octacosane	Anti-inflammatory, antifungal, antioxidant	[33]
2	Carboxylic acids	Hexadecanoic acid	Antibacterial, antioxidant	[22,47]
3	Alkanes	3-Ethyl-5-(2-ethylbutyl)octadecane	Antifungal	[34]
4	Alcohols	Behenic alcohol	N.R.	–
5	Alcohols	Octacosanol	Antioxidant, anti-inflammatory	[40]
6	Aldehydes	(E)-15-Heptadecenal	Antibacterial	[53]
7	Alcohols	Tetracosanol-1	Antibacterial	[39]
8	Carboxylic acids	(Z,Z,Z)-9,12,15-Octadecatrienoic acid	N.R.	–
9	Terpenes	Phytol	Antioxidant, anti-inflammatory, antimicrobial	[44]
10	Ketones	12-Tricosanone	N.R.	–
11	Alcohols	2-Hexyl-1-decanol	Antimicrobial	[36]
12	Alkenes	4,6,8-Trimethyl-1-nonene	N.R.	–
13	Terpenes	Squalene	Antioxidant, antibacterial	[46]
14	Alcohols	2-Octyl-1-decanol	Antimicrobial, antifungal	[37]
15	Alcohols	2-Octyl-1-dodecanol	N.R.	–
16	Alkanes	Heneicosane	Antimicrobial, antifungal	[31]
17	Alcohols	2-Hexyl-1-dodecanol	N.R.	–
18	Terpenes	2,6,11,15-Tetramethylhexadecane	Antibacterial	[43]
19	Phenyl derivatives	4-Ethoxybenzoic acid ethyl ester	N.R.	–
20	Alkenes	1-Nonadecene	N.R.	–
21	Alkanes	Heptadecane	Antioxidant, antimicrobial	[24]
22	Alkanes	Hexacosane	Antioxidant, antimicrobial	[32]
23	Alkanes	9-Octyl-hexacosane	N.R.	–
24	Aldehydes	Octadecanal	N.R.	–
25	Phenyl derivatives	Dibutyl phthalate	Antibacterial	[52]
26	Alkanes	Nonadecane	Antibacterial	[28]
27	Esters of carboxylic acids	Triacontanoic acid methyl ester	Antibacterial	[55]
28	Phenyl derivatives	Butylated hydroxytoluene	Antioxidant	[50]
29	Alkanes	Hexadecane	Antibacterial, antifungal, antioxidant	[25]
30	Alkanes	4,6-Dimethyldodecane	N.R.	–

31	Lactones	4,8,12,16-Tetramethylheptadecan-4-olide	N.R.	–
32	Esters of carboxylic acids	Octacosanoic acid methyl ester	N.R.	–
33	Alkanes	Octadecane	Anti-inflammatory, antifungal, antimicrobial	[26,27]
34	Esters of carboxylic acids	Hexacosanoic acid methyl ester	Antibacterial	[54]
35	Alkanes	Pentadecane	Antimicrobial	[23]
36	Esters of carboxylic acids	9,12,15-Octadecatrienoic acid methyl ester	N.R.	–
37	Alkenes	Cetene	Antimicrobial, antioxidant	[41]
38	Epoxides	Hexadecyl oxirane	N.R.	–
39	Carboxylic acids	Octadecanoic acid	Antibacterial, antioxidant	[48]
40	Terpenes	2,6,10,14-Tetramethylhexadecane	Antibacterial, antifungal	[42]
41	Terpenes	(E)-β-Farnesene	N.R.	–
42	Alkanes	2-Methyleicosane	N.R.	–
43	Esters of carboxylic acids	9,12-Octadecadienoic acid methyl ester	N.R.	–
44	Alkanes	2,6,10,14-Tetramethylpentadecane	N.R.	–
45	Phenyl derivatives	2,4-Bis(1,1-dimethylethyl)phenol	Antibacterial, anti-inflammatory	[51]
46	Quinoline derivatives	8-Amino-6-methoxy-5-[n-propoxy]quinoline	N.R.	–
47	Alkanes	9-Octyl-heptadecane	N.R.	–
48	Phenyl derivatives	2′,4′-Dimethoxy-3′-methylpropiophenone	N.R.	–
49	Alkanes	Hentriacontane	Antioxidant, antibacterial	[29,30]
50	Esters of carboxylic acids	Isopropyl myristate	N.R.	–
51	Carboxylic acids	Oleic acid	Antifungal	[49]

Note: The biological activities listed in this table are based on previously published reports for the corresponding compounds and are provided only for comparative and interpretative purposes. These data do not imply that the listed compounds were directly responsible for the antimicrobial activity observed in the present study. The contribution of individual compounds requires further confirmation through bioassay-guided fractionation, isolation, and mechanistic studies. Compounds were tentatively identified based on GC-MS library matching, and no authentic reference standards were used.

Abbreviations: GC-MS, gas chromatography–mass spectrometry; N.R., no relevant activity was reported in the literature sources reviewed for this table.
